# miR-21 Plays a Dual Role in Tumor Formation and Cytotoxic Response in Breast Tumors

**DOI:** 10.3390/cancers13040888

**Published:** 2021-02-20

**Authors:** Tu Dan, Anuradha A. Shastri, Ajay Palagani, Simone Buraschi, Thomas Neill, Jason E. Savage, Aastha Kapoor, Tiziana DeAngelis, Sankar Addya, Kevin Camphausen, Renato V. Iozzo, Nicole L. Simone

**Affiliations:** 1 Department of Radiation Oncology, Sidney Kimmel Cancer Center at Thomas Jefferson University, Philadelphia, PA 19107, USA; tu.dan@UTSouthwestern.edu (T.D.); axs791@jefferson.edu (A.A.S.); ajay.palagani@nih.gov (A.P.); Tiziana.DeAngelis@jefferson.edu (T.D.); 2 Anatomy and Cell Biology and the Translational Cellular Oncology Program, Sidney Kimmel Cancer Center, Department of Pathology, Sidney Kimmel Medical College, Thomas Jefferson University, Philadelphia, PA 19107, USA; simone.buraschi@jefferson.edu (S.B.); thomas.neill@jefferson.edu (T.N.); Aastha.Kapoor@jefferson.edu (A.K.); Renato.Iozzo@jefferson.edu (R.V.I.); 3 Radiation Oncology Branch, National Cancer Institute, Bethesda, MD 20892, USA; savagej@mail.nih.gov (J.E.S.); camphauk@mail.nih.gov (K.C.); 4 Department of Cancer Biology, Sidney Kimmel Cancer Center at Thomas Jefferson University, Philadelphia, PA 19107, USA; sankar.addya@jefferson.edu; 5 Department of Radiation Oncology, Sidney Kimmel Cancer Center at Thomas Jefferson University, 111 South 11th Street, Bodine Cancer Center, G-301G, Philadelphia, PA 19107, USA

**Keywords:** breast cancer, metastases, miR-21, apoptosis

## Abstract

**Simple Summary:**

miR-21 is an oncogenic microRNA that has been associated with breast tumor growth and metastasis in vitro and is also noted to be upregulated by cytotoxic stressors in model systems and in breast cancer patients who have undergone radiation. In the present study, our findings demonstrate the novel role of miR-21 in vivo for breast cancer initiation and metastases, and in sensitizing tumor cells to cytotoxic therapy by upregulating the FAS/FASL signaling pathway.

**Abstract:**

Breast cancer (BrCa) relies on specific microRNAs to drive disease progression. Oncogenic miR-21 is upregulated in many cancers, including BrCa, and is associated with poor survival and treatment resistance. We sought to determine the role of miR-21 in BrCa tumor initiation, progression and treatment response. In a triple-negative BrCa model, radiation exposure increased miR-21 in both primary tumor and metastases. In vitro, miR-21 knockdown decreased survival in all BrCa subtypes in the presence of radiation. The role of miR-21 in BrCa initiation was evaluated by implanting wild-type miR-21 BrCa cells into genetically engineered mouse models where miR-21 was intact, heterozygous or globally ablated. Tumors were unable to grow in the mammary fat pads of *miR-21^−/−^* mice, and grew in ~50% of *miR-21^+/−^* and 100% in *miR-21^+/+^* mice. The contribution of miR-21 to progression and metastases was tested by crossing *miR-21^−/−^* mice with mice that spontaneously develop BrCa. The global ablation of miR-21 significantly decreased the tumorigenesis and metastases of BrCa, while sensitizing tumors to radio- and chemotherapeutic agents via Fas/FasL-dependent apoptosis. Therefore, targeting miR-21 alone or in combination with various radio or cytotoxic therapies may represent novel and efficacious therapeutic modalities for the future treatment of BrCa patients.

## 1. Introduction

Breast cancer remains the most common malignancy in women in the United States, and while most women have favorable outcomes, a significant number of patients have poor responses to standard treatments [1]. Further delineation of the molecular underpinnings linking tumor initiation, progression and treatment resistance could lead to improved treatment modalities for breast cancer.

miRNAs can act as either oncogenes or tumor suppressors, and outside of these roles, they can also perturb processes such as epithelial–mesenchymal transition, vessel invasion and metastasis, and the evasion of apoptosis [2,3]. Recent data have implicated specific miRNAs in tumorigenesis and cancer progression, as well as resistance to treatment [4,5,6]. miR-21 is one of the most commonly upregulated miRNAs in breast cancer. It has prognostic implications as it has been closely linked to advanced tumor stage, lymph node metastasis, and decreased overall survival in multiple retrospective clinical studies [7,8,9,10]. Additionally, the upregulation of miR-21 has been consistently demonstrated with stress stimuli, such as free radical and DNA damage; thus, this microRNA could play a role in resistance to cytotoxic therapies, such as chemotherapy or radiotherapy, both of which are integral in the treatment of breast cancer [11,12,13,14,15].

While high levels of miR-21 portend a poor prognosis in breast cancer patients, there are no data suggesting that targeting miR-21 in vivo could improve disease progression in breast cancer and outcomes of cytotoxic therapies. Given its potential intersection among prognosis, tumorigenesis and treatment resistance, we sought to determine the role of miR-21 in breast cancer tumor development and progression using mouse models, including a newly created miR-21 knockout mouse. We utilized a model which mimics the spontaneous neoplastic transformation of the breast and the development of metastases in order to analyze the effects of miR-21 expression during disease progression [16]. Moreover, we examined the oncogenic effects of miR-21 and its response to DNA-damaging agents. We demonstrate that the loss of miR-21 decreases the tumorigenesis and metastasis of breast cancer, while sensitizing breast carcinomas to DNA-damaging agents through the Fas/FasL pathway.

## 2. Material and Methods

### 2.1. Cell Culture

4T1 cells, MDA-MB-468 cells, SKBR3 cells (ATCC) and MCF-7 (gifted by Dr. Karen Bussard) were verified with isoenzymology using the Authentikit system (ATCC, Manassas, VA, USA) and authenticated using STR profiling. All experiments were performed with mycoplasma-free cells. 4T1 cells were cultured in RPMI-1640, MDA-MB-468 in DMEM, MCF-7 in MEM and SKBR-3 in McCoy’s 5A media. All media were supplemented with 10% Fetal Bovine Serum and 1% Penicillin–Streptomycin solution (Invitrogen, Carlsbad, CA, USA). To obtain *miR-21^+/+^* and *miR-21^−/−^* cells for in vivo models, tumors were excised from *miR-21^+/+^;MMTV-PyMT* and *miR-21^−/−^*;*MMTV-PyMT* mice, minced, and digested by shaking in buffer containing 0.25% trypsin and 0.5 mg/mL collagenase in PBS. Samples were centrifuged for 10 min at 1500 rpm after which the supernatant was aspirated, and the tumor tissue was resuspended in media (DMEM, 10% Fetal Bovine Serum, 0.5% Penicillin–Streptomycin) and incubated at 37 °C. The *miR-21^+/+^;MMTV-PyMT* and *miR-21^−/−^*;*MMTV-PyMT* cells were cultured in media containing high-glucose DMEM, supplemented with 5% fetal bovine serum and sodium pyruvate (Life Technologies, Carlsbad, CA, USA).

### 2.2. Transfection and Cell Viability Assays

To determine the optimal concentration of miRvana miR-21 inhibitor for each cell line, the cell lines were transfected with Lipofectamine RNAiMAX transfection reagent as per the manufacturer’s guidelines (Thermofisher, Waltham, MA, USA), and 5 concentrations of the inhibitor or negative scramble control were applied: 0 nM, 1 nM, 30 nM, 60 nM and 90 nM. The final treatment concentrations chosen caused at least 50% miR-21 knockdown. Once the treatment concentrations of miR-21 inhibitor were determined, the cell lines were seeded at a density of 5 × 10^4^ cells in 24-well plates and transfected with 60 nM (MDA-MB-468), 90 nM (MCF-7) and 30 nM (SKBR-3) of miRvana miR-21-5p inhibitor or negative scramble control (Thermofisher, Waltham, MA, USA). Cell viability was determined with 3-(4, 5-dimethylthiozol-2-yl)-2, 5-diphenyltetrazolium bromide (MTT; Sigma Aldrich, St. Louis, MO, USA) using 0.5 mg/mL MTT and isopropyl alcohol:Triton X:HCl solubilization buffer.

### 2.3. Treatment for Autophagy Determination

*miR-21^+/+^;MMTV-PyMT* and *miR-21^−/−^*;*MMTV-PyMT* cells cultured as above were seeded at a density of 2 × 10^5^ cells/well in 6-well plates and treated at 90–95% confluency with 500 nM Bafilomycin (Sigma, St. Louis, MO, USA) for 6 h prior to lysis. DMSO (Thermofisher, Waltham, MA, USA) was used as the vehicle for Bafilomycin. The cells were then processed for analysis using western blot.

### 2.4. Mouse Models

The Institutional Animal Care and Use Committee approved all mouse studies.

#### 2.4.1. Breast Cancer Metastases Murine Model

Twenty BALB/c mice (female, 12-week-old, Charles River Laboratories, Wilmington, MA, USA) were orthotopically injected with 5 × 10^4^ 4T1 cells into the #4 mammary fat pad of the mice. Once the tumors measured ~100 mm^3^, 10 mice received sham irradiation and the other 10 received focal irradiation (6.5 Gy) to the primary tumor. The mice were monitored until the tumor volume reached 2000 mm^3^, and were then sacrificed, with primary tumors and lung metastases collected in RNA later (Qiagen, Germantown, MD, USA).

#### 2.4.2. *MMTV-PyMT* Orthotopic Mouse Model

*miR-21^+/+^, miR-21^+/−^* and *miR-21^−/−^* female FVB mice (6–8 weeks old) were orthotopically injected in the mammary fat pad with 10^6^ *miR-21^+/+^;MMTV-PyMT* cells. Post-injection, ten mice from each phenotype were monitored three times a week with tumor measurement for up to 10 weeks. Mice were euthanized and tumors collected when the largest primary tumor reached 1500 mm^3^, or the tumor burden reached 2000 mm^3^.

#### 2.4.3. *MMTV-PyMT* Genetically Engineered Mouse Model

Male *MMTV-PyMT* mice obtained from Jackson Laboratories were crossed with female *miR-21^+/+^* or *miR-21^−/−^* mice to generate homozygous male/female *miR-21^+/+^;MMTV-PyMT* and *miR-21^−/−^;MMTV-PyMT* mice. Mice were genotyped and only female *miR-21^+/+^* and *miR-21^−/−^*;*MMTV-PyMT* mice were used for experiments due to their mature mammary tissue. *miR-21^+/+^* and *miR-21^−/−^*;*MMTV-PyMT* mice were monitored for 18 weeks or until they reached humane endpoints for both primary and metastatic tumor formation. Primary tumors and lungs were collected at sacrifice for gross examination, and the number of metastases was counted. Additional tumor-bearing *miR-21^+/+^;MMTV-PyMT* and *miR-21^−/−^*;*MMTV-PyMT* mice were irradiated (6.5 Gy) when their tumors reached 1000 mm^3^, and were permitted to grow until the total tumor volume reached 2000 mm^3^. The tumors were divided into equal parts and placed in RNAlater or snap-frozen upon collection.

### 2.5. Human Sera

Human serum samples were obtained via IRB-approved protocol at the National Cancer Institute (02-C-0064) according to the protocol by Khoury et al. [17]. After signing informed consent, whole blood was obtained via venipuncture from women within one week of starting radiation and after the last fraction of radiation was delivered. The patients were treated with 50–60 Gy of radiation to the breast and/or regional lymph nodes. Whole blood was spun at 2500 rpm for 10 min and serum was aliquoted and frozen in liquid nitrogen.

### 2.6. Irradiation and Chemotherapeutic Treatments

Irradiation was delivered by a Pantak H-320 (320 kV); Precision X-Ray, N. Bradford, CT. Dosimetry was performed by an in-the-beam ionization chamber calibrated against a primary standard after making corrections for humidity, temperature and barometric pressure. For in vitro studies, cells were treated with either sham irradiation or 4 or 8 Gy for 72 h. For in vivo studies, the mice were shielded using a custom lead jig so that irradiation could be delivered to the primary tumor itself to a dose of 6.5 Gy. Transfected cells were also treated with chemotherapy agents: Doxorubicin (Adriamycin) (100 nM for MDA-MB-468, 360 nM for MCF-7, or 1 μM for SKBR3) and Taxol (Paclitaxel) (16 nM for MDA-MB-468, 68 nM for MCF-7, or 2 nM for SKBR-3) (Sigma Aldrich, St. Louis, MO, USA). The treatment concentrations of chemotherapy agents were based on previously determined concentrations that induced IC_50_ [18,19], or on the Genomics of Drug Sensitivity in Cancer database (www.cancerrxgene.org; accessed on 20 August 2020), and independently tested in our systems for final treatment concentrations.

### 2.7. Genotyping PCR, RNA Isolation and qRT-PCR

To genotype the mice for the genetically engineered and orthotopic mouse model as described above, genomic DNA from tail clips was isolated using the REDExtract-N-Amp Tissue PCR kit (Sigma-Aldrich). PCR was performed using the following primers: 5′-TTTATGACGCATTGCACACCCTC (forward), 5′-CACAGAGAAGTAAGCTTCCACCTGTTAAAG (WT reverse); 5′-AATAAGACTTATGAGATGGAGTCAGAAGGC (KO reverse). The expected product sizes were 492 and 578 bp for wild-type and knockout alleles, respectively.

Total RNA from cells, tissues or serum was isolated using Trizol reagent (Thermo Fisher Scientific, Waltham, MA, USA) using the Phenol–Chloroform RNA isolation technique. Following extraction, RNA concentration was measured (NanoDrop 1000, Thermo Scientific, Waltham, MA, USA), and 200 ng was reverse transcribed for both tissue and serum using the miScript II RT Kit (Qiagen, Germantown, MD, USA). Tissue miRNA expression was detected by qRT-PCR using the miScript SYBR Green PCR Kit (Qiagen, Germantown, MD, USA), and qPCR primer sets against hsa-miR-21-5p, mmu-miR-21-5p, FAS, controls UniSp6 and snord95 were performed in triplicate (Qiagen, Germantown, MD, USA) using an AB7500 fast system (Applied Biosystems, Life Technologies Corporation, Carlsbad, CA, USA) with data presented as 2-∆Ct. *miR-21^+/+^;MMTV-PyMT* and *miR-21^−/−^*;*MMTV-PyMT* cells were lysed with TRIzol Reagent (Life Technologies). RNA isolation was carried out with Direct-zol RNA Miniprep Kit as per the manufacturer’s protocol (Zymo Research, Irvine, CA, USA). Total RNA (1 μg) was annealed with oligo (dT12-18) primers, and cDNA was synthesized using SuperScript Reverse Transcriptase III (Life Technologies, Carlsbad, CA, USA). Gene-specific primer sets for M. musculus Map1lc3a and Actb were utilized. Gene expression analysis was performed on a Roche LightCycler 480-II and presented as 2-∆Ct.

### 2.8. Hematoxylin Eosin Staining

Paraffin-embedded tissue sections were stained with Harris’ hematoxylin (Sigma-Aldrich, St. Louis, MO, USA) and counterstained with eosin Y solution, alcoholic (Sigma-Aldrich, St. Louis, MO, USA), according to the manufacturer’s protocol.

#### 2.8.1. cDNA Profiling

Total RNA was isolated from irradiated *miR-21^−/−^;MMTV-PyMT* and *miR-21^+/+^*;*MMTV-PyMT* tumors, then quantified (Nanodrop ND-100 spectrophotometer), and quality assessed (Agilent 2200 TapeStation; Agilent Technologies, Palo Alto, CA, USA). Affymetrix gene chips (Mouse Gene 2.0-ST) were hybridized with 5 μg fragmented and biotin-labeled cDNA in 200 μL of hybridization cocktail. Target denaturation, followed by hybridization and staining, were done as per the methods used by Singh, J. et al. [20]. Differentially expressed gene lists were subjected to pathway analysis using IPA software, and significant genes were identified using the a priori selection criteria of absolute fold-change greater than or equal to 2, with a *p-*value < 0.05. The microarray data were deposited at GEO: GSE144773.

#### 2.8.2. Tissue Lysis, Western Blotting and Statistical Analysis

Tissues were lysed in T-PER tissue protein extraction reagent (Thermo Scientific, Waltham, MA, USA) as per the manufacturer’s instructions. Protein concentration was determined via a BCA assay, and SDS-PAGE-performed PVDF blots were probed with antibodies against FAS (Abcam, Cambridge, MA, USA), Caspase-3, 7, 8, 9, GAPDH (Cell Signaling Technology, Danvers, MA, USA), LC3, p62, β-Actin (Sigma, St. Louis, MO, USA), and were visualized with ECL substrate chemiluminescent detection reagent (Thermofisher, Waltham, MA, USA). ImageJ (NIH) software was used to quantify. *miR-21^+/+^;MMTV-PyMT* and *miR-21^−/−^*;*MMTV-PyMT* cells treated with 500 nM Bafilomycin were lysed using RIPA buffer for 25 min on ice, then resolved via SDS/PAGE. Proteins were then transferred to nitrocellulose membranes (Bio-Rad), immunoreacted with the indicated primary antibodies (GAPDH, LC3-I/-II, or p62) and developed with enhanced chemiluminescence (ThermoFisher Scientific). The membranes were then detected using an ImageQuant LAS-4000 (GE Healthcare, Chicago, IL, USA). A total of 3–5 biological replicates were performed for each experiment. The original western blotting figures can be found in Figure S4.

Statistics were assessed using a Student’s *t*-test for miRNA expression levels, one-way ANOVA followed by Dunnett’s post-test for mRNA and protein expression, and two-way ANOVA followed by Bonferroni post-test for miRNA expression levels and cell survival assays. Tumor-free survival was determined using the Mantel–Cox test to generate a Kaplan–Meier curve. The median tumor-free survival between the groups was compared and the significance was determined using a pre-specified α = 0.025 (0.05/2). Statistical analyses were done using GraphPad Prism software (GraphPad Software, La Jolla, CA, USA) or Sigma Stat (v3.10).

## 3. Results

### 3.1. miR-21 Expression Plays a Critical Role in Breast Cancer Growth, Metastases and Treatment

**Figure 1:**
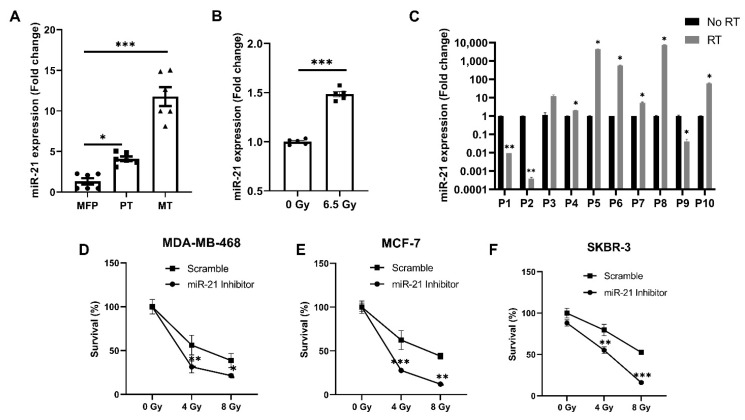
Stress-activated miR-21 plays a critical role in breast cancer growth and metastases. (**A**) miR-21 expression was increased as the breast cancer progressed. Basal levels of miR-21 were seen in the normal fat pad, the orthotopic primary tumor showed increased expression of miR-21, and the maximum increase in miR-21 was seen in lung metastases (*n* = 2 mice/group; ●—Individual replicates for normal mammary fat pad (MFP), ▪—Individual replicates of primary tumor (PT) ▲—Individual replicates of Metastases (MT). (**B**) Radiation-caused stress induced increases in miR-21 expression in the primary tumor (*n* = 2 mice/group; ●—Individual replicates for primary tumor without radiation, ▪—Individual replicates of primary tumor irradiated with dose 6.5 Gy). (**C**) miR-21 expression was increased in human serum post-radiation (*n* = 10). (**D**) Knockdown of miR-21 with a specific inhibitor sensitized MDA-MB-468, MCF-7 (**E**), and SKBR3 (**F**) cells to increasing doses of radiation over a total treatment period of 72 h (*n* = 3/group). For (**A**–**C**), mean ± SEM with ***, ** are significantly different with *p* < 0.001 or <0.01 as determined by one-way ANOVA or student’s *t*-test. For (**D**–**F**), mean ± SD with ***, **, * are significantly different with *p* < 0.001 or < 0.01 or 0.05 as determined by one-way ANOVA or student’s *t*-test.

As an increased expression of miR-21 is implicated in the poor prognosis and progression of several types of cancer and treatment resistance, we used an in vivo model for the first time to evaluate this in triple-negative breast cancer. In this 4T1 model, the expression of miR-21 was increased ~4-fold in primary tumors and ~11.8-fold in the metastases compared to normal mammary fat pad tissue (MFP) (Figure 1A). Prior studies have shown miR-21 to play a role in radiation resistance, so primary tumors were also evaluated for miR-21 expression in the presence or absence of radiation [21,22]. Tumors treated with radiation showed a ~1.5-fold increase in miR-21 expression compared to the untreated tumors (Figure 1B). Next, we validated these animal experiments by determining the miR-21 levels in human sera collected from breast cancer patients (*n* = 10) before or after a six-week course of radiation. We discovered that radiation caused an increase in the circulating levels of miR-21 in the sera of irradiated patients (Figure 1C). As miR-21 increased with the progression of disease, and also increased with radiation in a syngeneic murine model and was noted to be consistently upregulated in patient samples, we wanted to determine if miR-21 knockdown could increase radiation sensitivity in breast cancer cells of all subtypes. miR-21 inhibition caused significant inhibition of tumor growth compared to scramble control in the triple-negative cell line MDA-MB-468 (Figure 1D, *p* < 0.05), the estrogen-positive MCF-7 line (Figure 1E, *p* < 0.001) and the Her-2-neu-positive breast cancer cell line SKBR-3 (Figure 1F, *p* < 0.05). Collectively, these results indicate that miR-21 is associated with breast cancer progression and the stress response evoked by radiation treatment in murine and human models. Importantly, the loss of miR-21 significantly increases radiation sensitivity in in vitro breast cancer models.

### 3.2. Loss of miR-21 Protects Mice from Breast Cancer Initiation: Orthotopic Model

**Figure 2:**
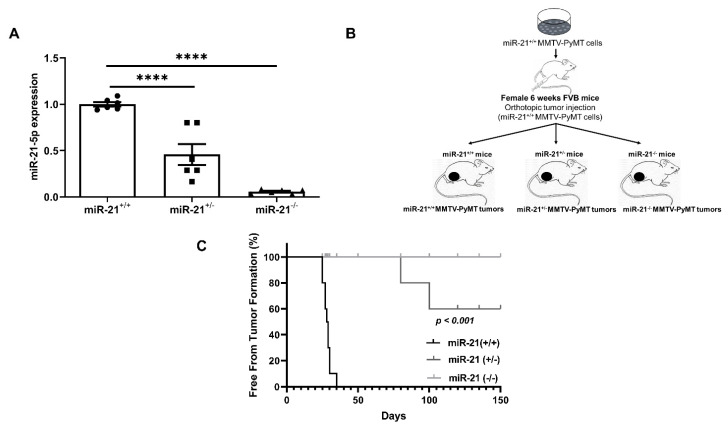
Genetic deletion of miR-21 protects against breast tumor formation. In the orthotopic model, 1 × 10^6^ MMTV-PyMT cells were injected into the mammary fat pad of *miR-21^+/+^, miR-21^+/−^* and *miR-21^−/−^* (*n* = 10 mice/group). (**A**) miR-21 expression in mammary fat pads of *miR-21^+/+^, miR-21^+/−^* and *miR-21^−/−^*, determined by one-way ANOVA and represented as mean ± SEM, (****—*p* < 0.0001, *n* = 2 mice/group; ●—Individual replicates of *miR-21^+/+^* tumors, ▪—Individual replicates of *miR-21^+/−^* tumor, ▲—Individual replicates of *miR-21^−/−^* tumors). (**B**) Schema. (**C**) Tumor formation was noted in all *miR-21^+/+^* and 45% of *miR-21^+/−^* mice, but the *miR-21^−/−^* had 0% tumor formation at 150 days determined by Mantel–Cox test (*p* < 0.001, *n* = 10 mice/group).

Although miR-21 is noted to play a role in breast cancer, its ability to initiate cancer has not been explored. To understand the effect of miR-21 on tumor initiation, we isolated miR-21 wild-type breast carcinoma cells from MMTV-PyMT mice, in which MMTV-LTR drives the expression of mammary gland-specific polyomavirus middle T-antigen, leading to the rapid development of highly metastatic tumors [23]. We then orthotopically injected them into the mammary fat pads of mice with three distinct genotypes, namely, *miR-21^+/+^*, *miR-21^+/−^* and *miR-21^−/−^* [24] (Figure 2B). The expression of miR-21 was verified in each genotype, and revealed a ~50% and ~95% reduction in *miR-21^+/−^* mice and *miR-21^−/−^* mice, respectively (Figure 2A). We found that five weeks after injection, 100% of the *miR-21^+/+^* mice developed tumors. In contrast, by the end of the study at 150 days, only ~45% of the miR-21^+/−^ mice developed tumors, and none of miR-21^−/−^ mice had detectable neoplasms (Figure 2C). Collectively, these findings demonstrate that the lack of miR-21 in the tumor microenvironment prevented the growth of tumors, suggesting that miR-21 may play an important role in tumor initiation.

To understand the mechanism by which a lack of miR-21 in the tumor microenvironment prevents tumor growth, *miR-21^+/+^;MMTV-PyMT* and *miR-21^−/−^*;*MMTV-PyMT* cells isolated from MMTV tumors were analyzed for expression and protein levels of autophagy markers. The cells were analyzed for the expression of *MAP1LC3A* using qRT-PCR. The MMTV-PyMT cells were also treated with Bafilomycin A1, which blocks the fusion of autophagosomes and lysosomes, and we analyzed its effect on the levels of p62 and LC3-I/II using western blots. We observed that there was no difference in the mRNA expression of *MAP1LC3A* between the *miR-21^+/+^;MMTV-PyMT* and *miR-21^−/−^*;*MMTV-PyMT* cells. Basal levels of LC3-II were significantly higher (*p* < 0.001) in the *miR-21^−/−^* cells. These findings showing increased autophagy were validated by treating the cells with Bafilomycin A1. We observed that the basal levels of p62 were reduced in *miR-21^−/−^* cells compared to *miR-21^+/+^* cells. Bafilomycin caused a significant increase in both p62 and LC3-II (*p* < 0.001) in the *miR-21^−/−^* cells, which indicates an increase in autophagic flux (Figure S3).

### 3.3. Loss of miR-21 Delays Tumor Growth and Reduces Metastases: Genetically Engineered Model

**Figure 3:**
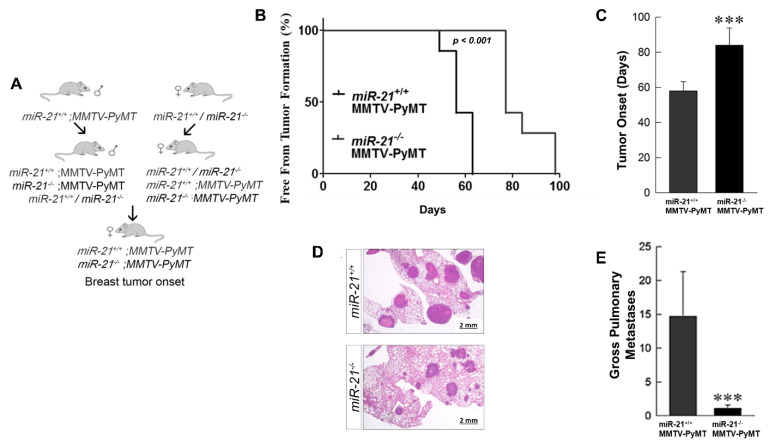
Genetic deletion of miR-21 delays tumor growth and reduces metastases in MMTV-PyMT mice. (**A**) Schema. (**B**,**C**) In crossing MMTV-PyMT mice with *miR-21^+/+^* and *miR-21^−/−^* mice the *miR-21^−/−^;MMTV-PyMT* mice were noted to have a delay in tumor formation of 4 weeks compared with the *miR-21^+/+^*;*MMTV-PyMT* mice, which was a statistically significant delay determined by Mantel–Cox test (3B) and student *t*-test (3C) (***-*p* < 0.001, *n* = 3 mice/group). (**D**) H&E staining of lung metastases in *miR-21^+/+^;MMTV-PyMT* and *miR-21^−/−^*;*MMTV-PyMT* at 16 and 18 weeks after birth. (**E**) Total number of external lung metastases in the *miR-21^+/+^* and *miR-21^−/−^*;*MMTV-PyMT* mice. Tumor regrowth delay was determined using the Mantel–Cox test to generate a Kaplan–Meier curve. The overall tumor uptake ratio for the injected groups was compared among the groups and significance was determined using a pre-specified α = 0.025 (0.05/2).

To evaluate if the loss of miR-21 could prevent or slow tumor progression in MMTV-PyMT mice that typically demonstrate rapid tumor formation and progression, we crossed our *miR-21^−/−^* and *miR-21^+/+^* mice with the MMTV-PyMT mice (Figure 3A). Notably, the *miR-21^−/−^;MMTV-PyMT* mice showed a delayed formation of tumors, with initiation at ~11 weeks compared to the *miR-21^+/+^*;*MMTV-PyMT* mice that developed tumors at ~8 weeks, *p* < 0.001 (Figure 3B,C).

Since we found miR-21 levels to be elevated in breast cancer metastases (cfr. Figure 1A), we investigated the contribution of miR-21 to metastasis. We found that the lack of miR-21 reduced metastases with a decrease in average size (Figure 3D). Gross examination of the total pulmonary metastases showed a significant reduction in the mice lacking miR-21 (Figure 3E, *p* < 0.05), with an average of 15 metastases in the *miR-21^+/+^*;*MMTV-PyMT*, and ~1 metastases in the *miR-21^−/−^*;*MMTV-PyMT* mice. Therefore, the loss of miR-21 in this model causes a significant delay in tumor progression and metastases despite the aggressive MMTV phenotype.

### 3.4. Radiation Increases Apoptosis in MMTV-PyMT Tumors Lacking miR-21

**Figure 4:**
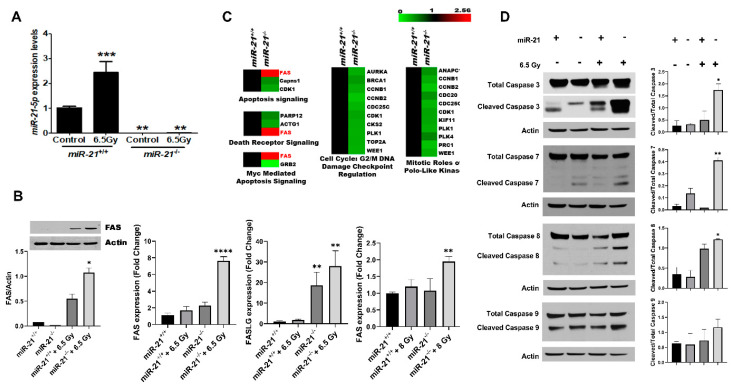
Apoptosis and death receptor signaling pathways contribute to the radiation-sensitive phenotype of the *miR-21^−/−^* mice. (**A**) To investigate the underlying mechanism of radiation sensitivity conferred by loss of miR-21, tumors generated from *miR-21^−/−^;MMTV-PyMT* and *miR-21^+/+^;MMTV-PyMT* mice were exposed to 6.5 Gy irradiation, and collected after 24 h. Irradiation caused a 2.5-fold induction of *miR-21* in the tumors derived from *miR-21^+/+^;MMTV-PyMT* mice, whereas no increase was noted in the *miR-21^−/−^* tumors determined using one-way ANOVA (***-*p* < 0.001, **-*p* < 0.01, *n* = 3/group). (**B**) As *FAS* and *FASL* are known targets of miR-21, the inverse correlation in the expression of *miR-21* and *FAS* was confirmed transcriptionally and at the protein level for Fas in tumors derived from *miR-21^−/−^;MMTV-PyMT* and *miR-21^+/+^;MMTV-PyMT* mice exposed or not to 6.5 Gy. MDA-MB-468 cells were transfected with 60 nM miR-21 inhibitor or scramble with or without 8 Gy. miR-21 inhibition combined with 8 Gy radiation increased FAS expression by 95% (*p* < 0.01) compared to control, and 63% (*p* < 0.05) compared control tumors treated with 8 Gy. miR-21 inhibition also caused significant increases in the expression of FASLG (*p* < 0.01) in tumors, and FAS (*p* < 0.01) in MDA-MB-468 cells treated with 8 Gy and miR-21 inhibitor, determined using one-way ANOVA (****-*p* < 0.000, **-*p* < 0.01, *-*p* < 0.05, *n* = 3/group). (**C**) Ingenuity pathway analysis revealed the activation of apoptosis and death receptor signaling pathways as top canonical pathways. (**D**) Twenty-four hours after radiation, the activation of the caspase 3, 7, 8, and 9 signaling pathways was evaluated in *miR-21^−/−^* and *miR-21^+/+^* tumors by western blot and quantified. Significant upregulation of cleaved caspases was noted in the *miR-21^−/−^* tumors (*n* = 3/group). In detail, caspase 3 was increased by 90%, 7 by 80%, 8 by 70% and 9 by 50% (**-*p* < 0.01, *-*p* < 0.05. Data represent the difference in means represented as mean ± SEM of three independent mouse tumor tissues normalized to housekeeping genes *snoRNA 95, snoRNA96A* miRNA, UniSp6, *GAPDH* mRNA and actin; *p* < 0.001 or <0.01 as determined by one-way ANOVA with Dunnett’s Post-test.

To evaluate the differential effect of radiation therapy in breast tumors in vivo, tumors derived from the *miR-21^+/+^;MMTV-PyMT* and *miR-21^−/−^*;*MMTV-PyMT* models were treated with radiation. The wild-type tumors had a ~2.5-fold increase in miR-21 evoked by 6.5 Gy (Figure 4A, *p* < 0.001). In contrast, no expression was noted in the comparably irradiated *miR-21^−/−^;MMTV-PyMT* tumors. To elucidate the underlying radiation-induced stress response mechanism, cDNA arrays were performed and revealed 460 differentially expressed genes in the irradiated breast tumors from *miR-21^−/−^* versus *miR-21^+/+^; MMTV-PyMT* mice (GEO: GSE144773). Ingenuity pathway analysis of the 80 genes upregulated in the absence of miR-21 (Figure S2) revealed the activation of apoptosis, death receptor signaling, and the Myc-mediated apoptosis signaling pathway. We found that FAS was common to all top pathways (Figure 4B). Notably, FAS is an apoptosis-inducing protein and an established target of miR-21 [25,26,27], known to be upregulated post-radiation in several types of cancer [28,29,30] with a critical role in radiation-induced cell death [25,26,27].

Next, to validate the transcriptomic findings, tumor samples were queried for FAS and FASL, both being known targets of miR-21, and both were notably induced by radiation in the *miR-21^−/−^* tumors at the mRNA and protein levels (*p* < 0.001, Figure 4C). This finding was verified in vitro with MDA-MB-468 cells transfected with miR-21 inhibitor. We found that following radiation there was a ~63% increase (*p* < 0.05) in FAS expression vis-à-vis control cells treated with radiation, and a ~95% increase (*p* < 0.01) compared to control (Figure 4D). Given that irradiation induced FAS and FASL in breast tumors lacking miR-21, we evaluated the activation of the caspase cascade (Figure 4F). We found a ~3.2-fold increase in cleaved Caspase-3 (*p* < 0.05), a ~3.6-fold increase in cleaved Caspase-7 (*p* < 0.01), and a significant increase in cleaved Caspase-8 and -9 (*p* < 0.01 and *p* < 0.05, respectively). Collectively, our findings expand our current knowledge of this onco-miR, and suggest that miR-21 could be a novel therapeutic target for apoptotic induction during radiotherapy.

### 3.5. The Inhibition of miR-21 Sensitizes Breast Cancer Cells to Cytotoxic Therapies

**Figure 5:**
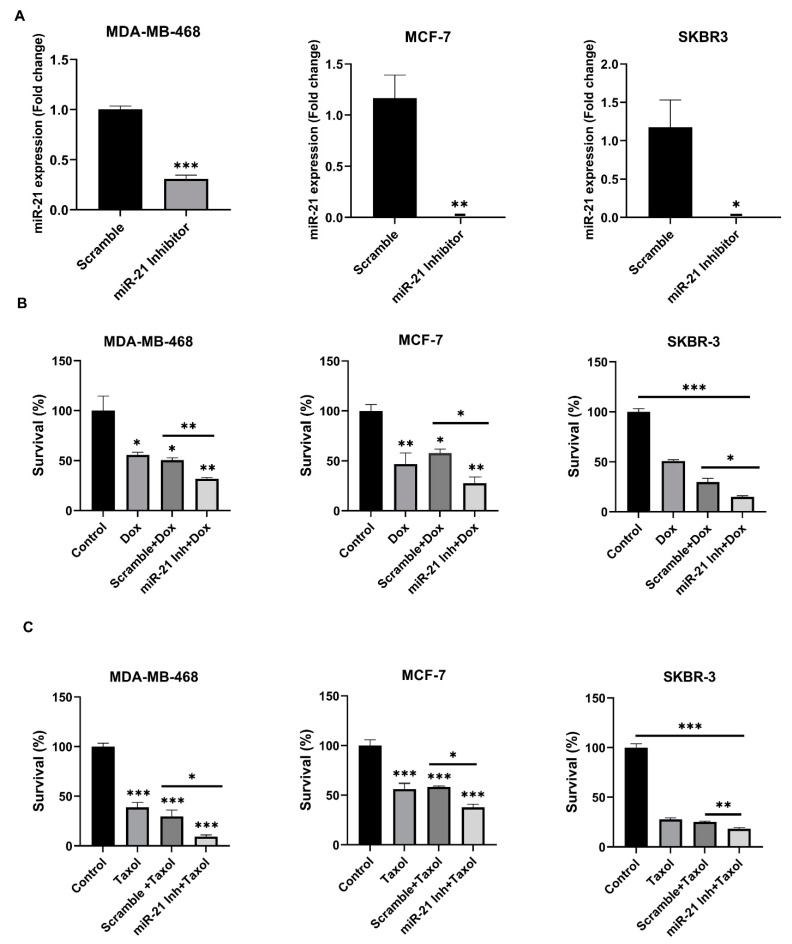
Chemosensitizing effect of *miR-21^−/−^* in breast cancer cells. Knockdown of miR-21 by transfecting MDA-MB-468, MCF-7 and SKBR-3 cells with miR-21 inhibitor sensitizes them to chemotherapy drugs; (**A**) 90 nM miR-21 inhibitor causes a ~70% decrease in miR-21 expression in MDA-MB-468 cells compared to scramble, while 90 nM in MCF-7, and 30 nM in SKBR-3 cells, caused almost complete miR-21 inhibition compared to scramble. (**B**) At 48 h post-treatment, miR-21 inhibition combined with doxorubicin caused a ~40%, ~50%, and ~50% decrease in cell survival of MDA-MB-468, MCF-7, and SKBR-3, respectively, compared to scramble control. (**C**) At 48 h post-treatment, miR-21 inhibition combined with taxol caused a ~70%, ~53% and ~28% decrease in the cell survival of MDA-MB-468, MCF-7, and SKBR-3, respectively compared to scramble control. All the in vitro experiments were done in triplicates or quadruplets (*n* = 3/group), *** *p* < 0.001 or ** *p* < 0.01 or * *p* < 0.05, determined by two-way ANOVA with Bonferroni post-test or student’s *t*-test.

To determine if the potential therapeutic advantage of miR-21 inhibition may also apply to other cytotoxic therapies, we evaluated the role of miR-21 in chemotherapeutic resistance. A cell line representing each breast cancer subtype was chosen and treated with Doxorubicin or Taxol in the presence of either scramble control or miR-21 inhibitor. First, we confirmed that transfection did not affect cell survival (Figure S1) and caused a decrease in miR-21 expression of ~69% in MDA-MB-468, as well as a >95% reduction in MCF-7 and SKBR-3 cells (Figure 5A). Next, we found that Doxorubicin alone caused a 44% (*p* < 0.05), 53% (*p* < 0.01) and 49% (*p* < 0.001) decrease in cell survival in MDA-MB-468, MCF-7 and SKBR-3 cells, respectively (Figure 5B). Notably, the combination of doxorubicin and miR-21 inhibitor further increased the cell death effects, with a decrease in cell survival to 68% (*p* < 0.01), 72% (*p* < 0.05) and 85% (*p* < 0.05) in the three cell lines, respectively (Figure 5B). Similarly, compared to control, Taxol alone caused a 61% (*p* < 0.001), 44% (*p* < 0.001) and 72% (*p* < 0.0001) decrease in cell survival in the MDA-MB-468, MCF-7, and SKBR-3 cells, respectively, and when combined with the miR-21 inhibitor, we saw a further decrease in cell survival of 91% (*p* < 0.05), 62% (*p* < 0.05) and 82% (*p* < 0.01) in the three cell lines, respectively (Figure 5C). Taken together, these results indicate that targeting miR-21 may increase the therapeutic efficacy of breast cancer therapies.

## 4. Discussion

miR-21 is an onco-miR [31,32,33] that has been associated with breast tumor growth and metastasis in vitro [34,35,36,37], and is noted to be upregulated by cytotoxic stressors in model systems [38,39,40] and in breast cancer patients who have undergone radiation [38,41]. In the present study, we demonstrate a novel role of miR-21 in vivo for breast cancer initiation and metastases, and in sensitizing tumor cells to cytotoxic therapy by upregulating the FAS/FASL signaling pathway.

Based on previously published results elaborating the role of miR-21, and our own data, we used *miR-21^−/−^* mice that we generated in our laboratory, and demonstrated for the first time that tumor uptake of orthotopic breast carcinoma allografts is unable to occur with a genetic deletion of both alleles of miR-21. Moreover, we demonstrated that a lack of miR-21 protects the mice from tumor formation as compared to the development seen in the mice with the presence of either one or two copies of the allele (*miR-21^+/−^*, *miR-21^+/+^*). These data imply that miR-21 expression in the host tumor microenvironment is necessary for tumor formation. The lack of tumor uptake in the host tumor environment of *miR-21^−/−^* mice may be related to multiple mechanisms. We hypothesize that a lack of tumor growth may be influenced by a decrease in pro-inflammatory markers, or an induction of autophagy. Using lung cancer models, others have shown that miR-21 alters the tumor microenvironment by increasing the secretion of the pro-inflammatory cytokines TNF- α and IL-6, through the activation of NF-κB, which decreases the number of CD8+ TIL and promotes the polarization of M2 macrophages [42]. Our data using *miR-21^−/−^*;*MMTV-PyMT* tumors demonstrated an increase in autophagic flux in *miR-21^−/−^* cells (Figure S3). This suggests the lack of tumor initiation in the *miR-21^−/−^* mice, and the delayed tumorigenic response and metastasis in the *miR-21^−/−^; MMTV-PyMT* model could be the direct result of an unfavorable tumor microenvironment characterized by augmented autophagic activity.

We also showed a delay in tumor formation using our genetically engineered *miR-21^−/−^;MMTV-PyMT* mouse model. Although this is a novel finding in breast cancer, Ma. et al. [43] and Li et al. [44] have shown a similar delay in a skin carcinogenesis model. We also evaluated the contribution of miR-21 to metastatic diseases in this GEMM system, and demonstrated that *miR-21^−/−^* mice have decreased metastases in the MMTV-PyMT model compared to *miR-21^+/+^*; *MMTV-PyMT* mice. Taken together, we postulate that these effects may be related to a change in the extracellular matrix that occurs with the deletion of miR-21. This could mechanistically explain the lack of primary tumor engraftment, as well as the tumor progression from the primary site to metastatic disease. It may also underlie the tumor-suppressive benefits following miR-21 loss and/or inhibition.

As we had noted a reflexive increase in miR-21 after exposure to radiation, and others have shown a link between miR-21 and radiation resistance in different models of cancers and that knocking down miR-21 creates a radiosensitive phenotype in vitro [11,21,22,45,46], we used our model to determine a mechanistic connection. Using in vitro invasion and wound healing assays, other researchers have shown that the inhibition of miR-21 using miR-21 inhibitors prevented or reduced cell migration and invasiveness. This may indicate that targeting miR-21 in vivo would reduce epithelial–mesenchymal transition [47,48,49]. Pathways analysis of primary breast tumors lacking miR-21 and exposed to ionizing radiation revealed apoptosis and death receptor signaling, specifically FAS/FASL signaling, to be the key signaling nodes in breast cancer treatment response in the *miR-21^−/−^* model. The ability to modulate FAS could be important, as it is induced by radiation, and the knockdown of FAS resulted in the increased survival of breast cancer cells following radiation [29]. Moreover, Wu et al. [10] and others [25,26,27,50] have shown an association of FASL with miR-21 expression in breast cancer. Therefore, our data suggest that *miR-21^−/−^* has the capability to sensitize MMTV-PyMT tumors to radiation therapy, and that this is accomplished by the upregulation of FAS/FASL signaling, activating the caspase cascade and resulting in increased apoptosis due to radiation in this model.

Additionally, using in vitro studies, we determined that inhibiting miR-21 expression also increased cell death in ER-positive, Her-2-neu-positive and triple-negative breast cancer cells when either radiation therapy or chemotherapies were used. These data suggest a therapeutic benefit to decreasing miR-21 in breast cancer. Others have demonstrated the chemosensitizing effect of downregulating miR-21 in diverse cancer types, including glioblastoma multiforme, cervical cancer, and lung cancer [7,45,51,52,53,54].

## 5. Conclusions

The present study demonstrates that miR-21 plays a role in breast cancer initiation, progression and therapeutic response. In a BrCA model, radiation exposure increases miR-21 in both the primary tumor and metastases. In vitro, miR-21 knockdown decreases survival in all BrCa subtypes in the presence of radiation and chemotherapeutic agents. The presence of miR-21 in the tumor microenvironment plays a role in the ability of BrCa tumors to initiate. miR-21 in the tumor contributes to progression and metastases, while sensitizing tumors to radio- and chemotherapeutic agents by Fas/FasL-dependent apoptosis. Targeting miR-21 alone or in combination with various radio or cytotoxic therapies may represent a novel therapeutic strategy for the treatment of BrCa patients.

## Data Availability

The data that support the findings of this study are available from the corresponding author upon reasonable request. The cDNA microarray data were deposited at GEO: GSE144773.

## Supplementary Materials

The following are available online at https://www.mdpi.com/2072-6694/13/4/888/s1, Figure S1: MDA-MB-468, MCF-7 and SKBR-3 treated with miRvana miR-21 inhibitor or scramble showed little or no decrease in cell survival compared to control, Figure S2: Heat maps of an Affymetrix gene array showing two-fold or greater changes in the gene expression of different gene signaling categories (*p* < 0.05), Figure S3: In our tumor cells isolated from *miR-21^−/−^*; MMTV-PyMT mice tumor we found no difference in expression of Map1lc3a (top left), but higher amounts of LC3-II (bottom right; *p* < 0.001), which indicates increased autophagy. We also found an increase of p62 (top right; *p* < 0.001) and LC3 (bottom right; *p* < 0.001) by blocking fusion of autophagosomes and lysosomes with Bafilomycin A1 in the *miR-21^−/−^* cells indicative of increased autophagic flux, Figure S4: The original western blotting figures.
