# Supplementary material for: miR-21 Plays a Dual Role in Tumor Formation and Cytotoxic Response in Breast Tumors
Source: Cancers (Basel). 2021 Feb 20;13(4):888. doi: 10.3390/cancers13040888 (PMC7924198; doi:10.3390/cancers13040888)
Supplement: Supplementary file 1 [file cancers-13-00888-s001.zip › cancers-1095370-supplementary.docx]

Supplemental Materials

miR-21 Plays A Dual Role in Tumor Formation and Cytotoxic Response in Breast Tumors

Tu Dan, Anuradha A. Shastri, Ajay Palagani, Simone Buraschi, Thomas Neill, Jason E. Savage, Aastha Kapoor, Tiziana DeAngelis, Sankar Addya, Kevin Camphausen, Renato V. Iozzo and Nicole L. Simone


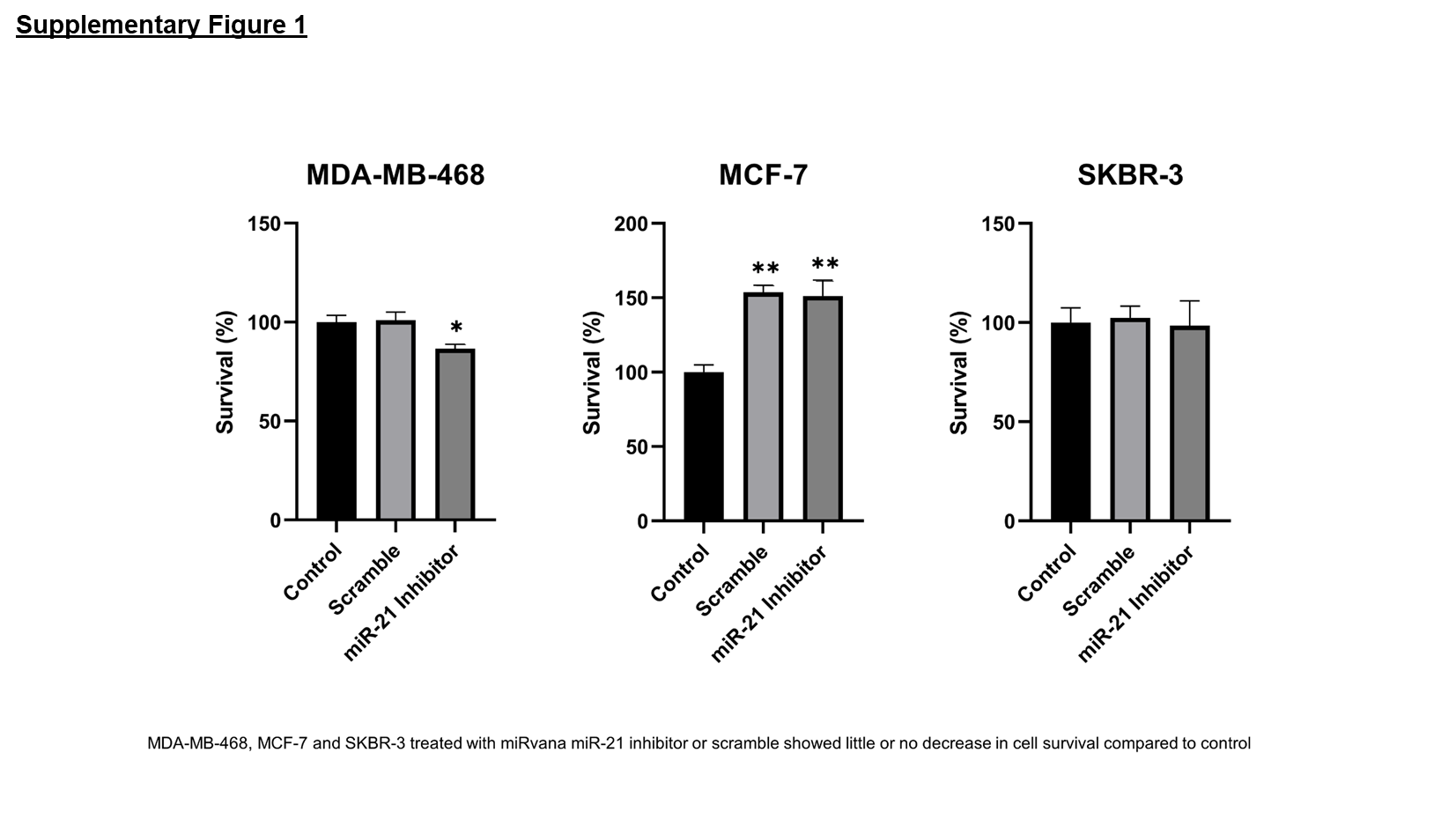


**Figure S1:** MDA-MB-468, MCF-7 and SKBR-3 treated with miRvana miR-21 inhibitor or scramble showed little or no decrease in cell survival compared to control.


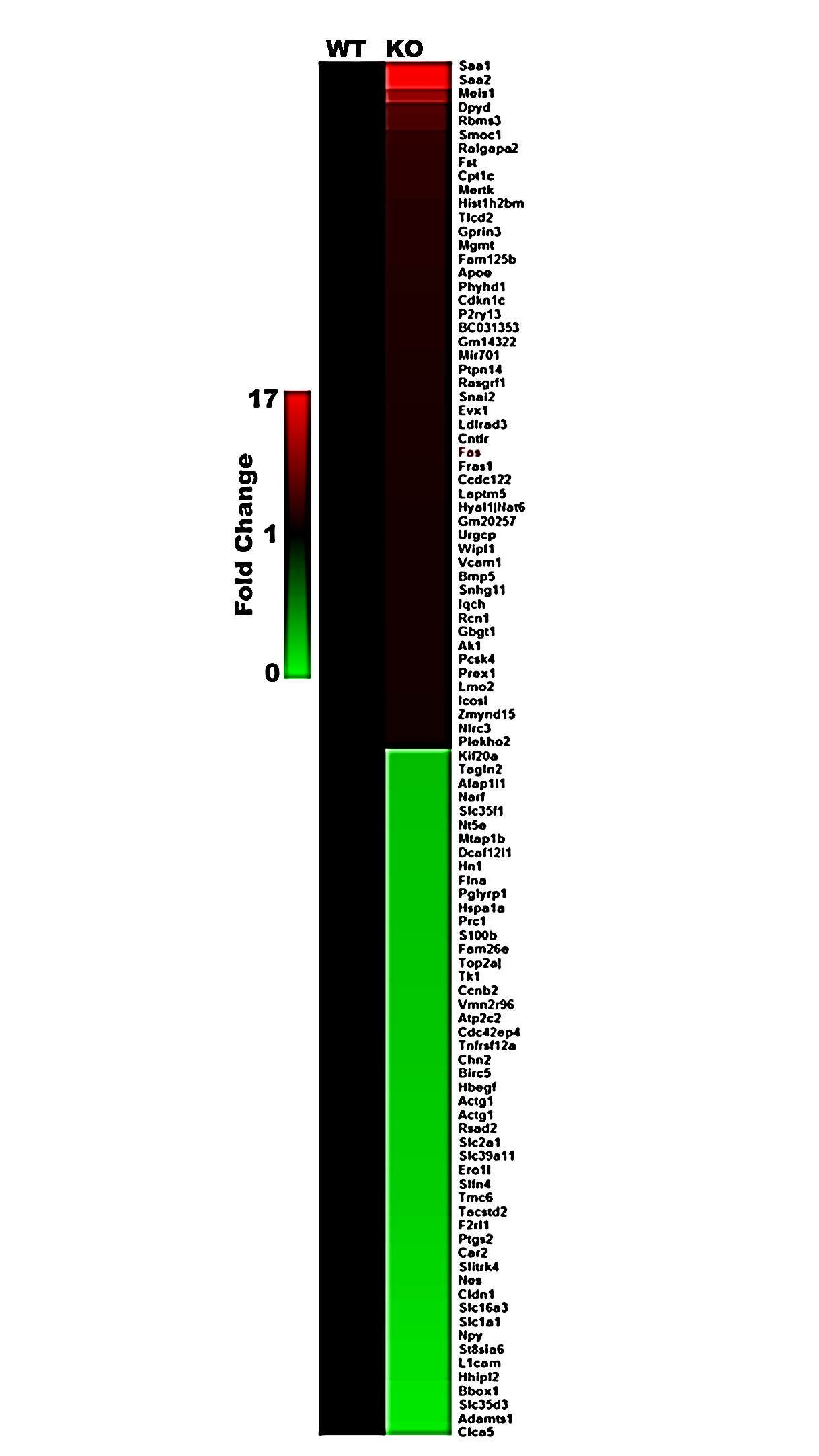


**Figure S2:** Heat maps of an Affymetrix gene array showing two-fold or greater changes in the gene expression of different gene signaling categories (*p* < 0.05).


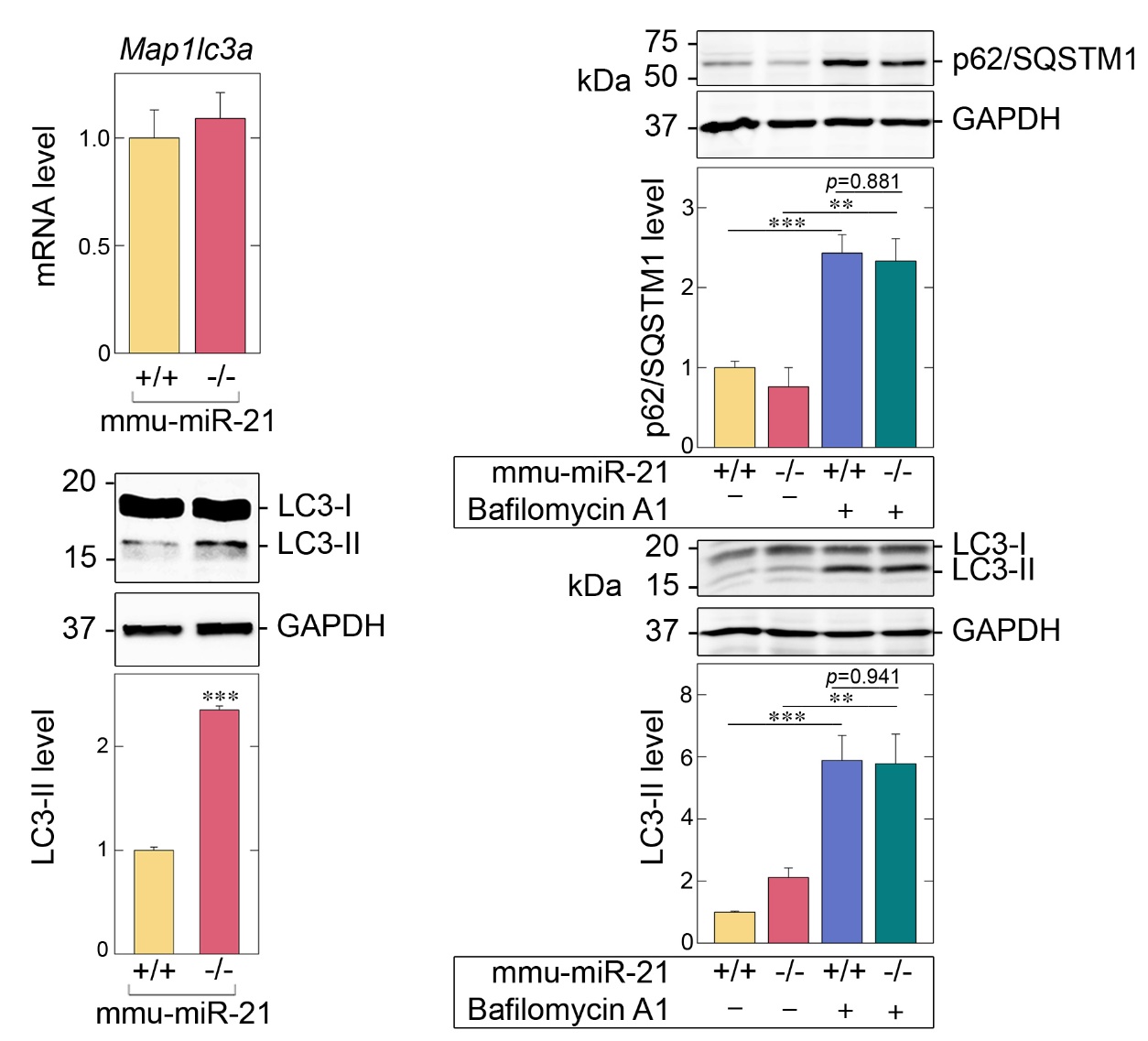


**Figure S3:** In our tumor cells isolated from miR-21*^−/−^*; MMTV-PyMT mice tumor we found no difference in expression of Map1lc3a (top left), but higher amounts of LC3-II (bottom right; *p* < 0.001), which indicates increased autophagy. We also found an increase of p62 (top right; *p* < 0.001) and LC3 (bottom right; *p* < 0.001) by blocking fusion of autophagosomes and lysosomes with Bafilomycin A1 in the miR-21*^−/−^* cells indicative of increased autophagic flux.
