# Supplementary material for: miR-21 Plays a Dual Role in Tumor Formation and Cytotoxic Response in Breast Tumors
Source: Cancers (Basel). 2021 Feb 20;13(4):888. doi: 10.3390/cancers13040888 (PMC7924198; doi:10.3390/cancers13040888)
Supplement: Supplementary file 1 [file cancers-13-00888-s001.zip › Figure S4-Original Western Blot Figures.pptx]

## Slide 1
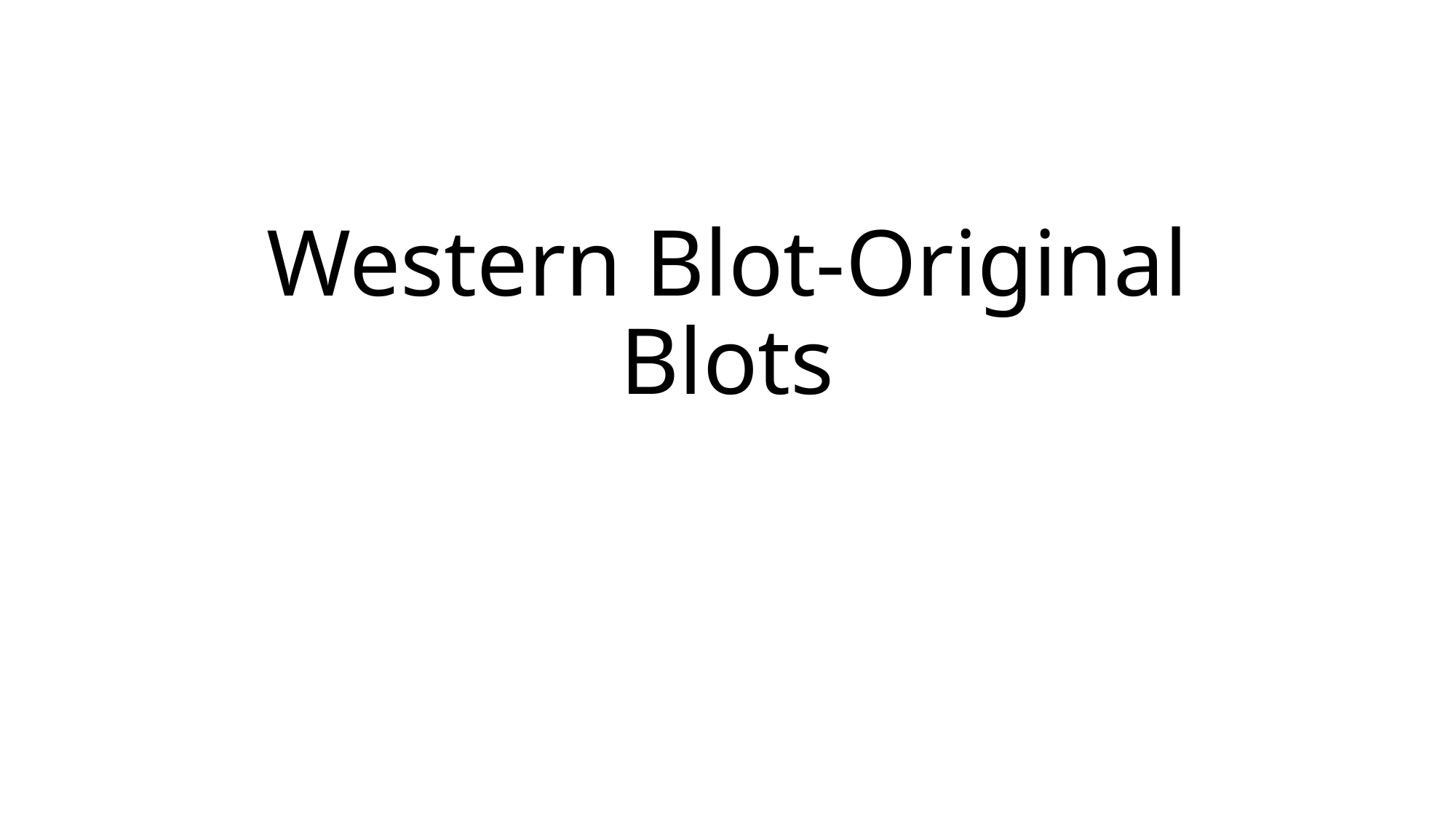

# Western Blot-Original Blots

## Slide 2
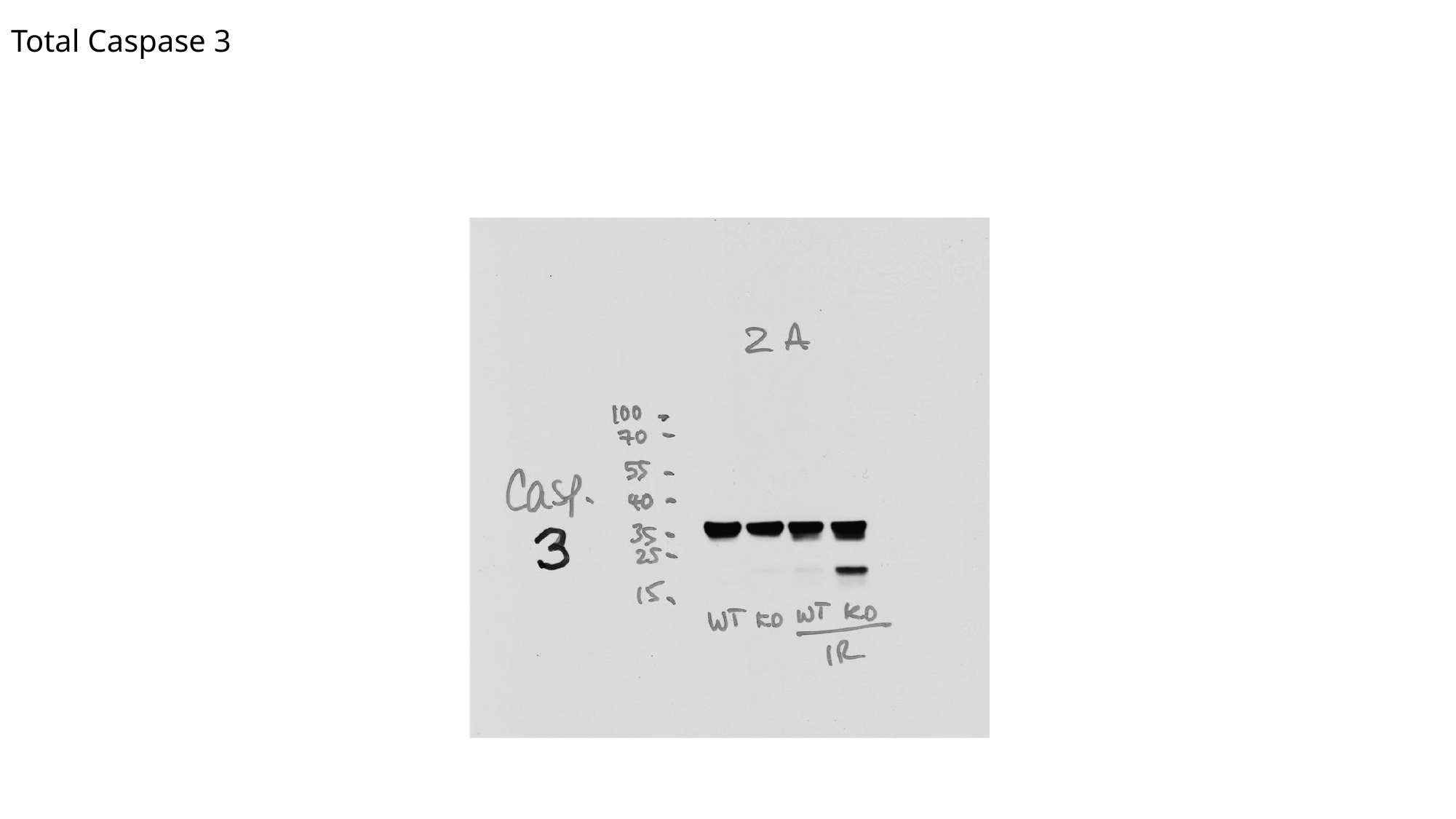

# Total Caspase 3

## Slide 3
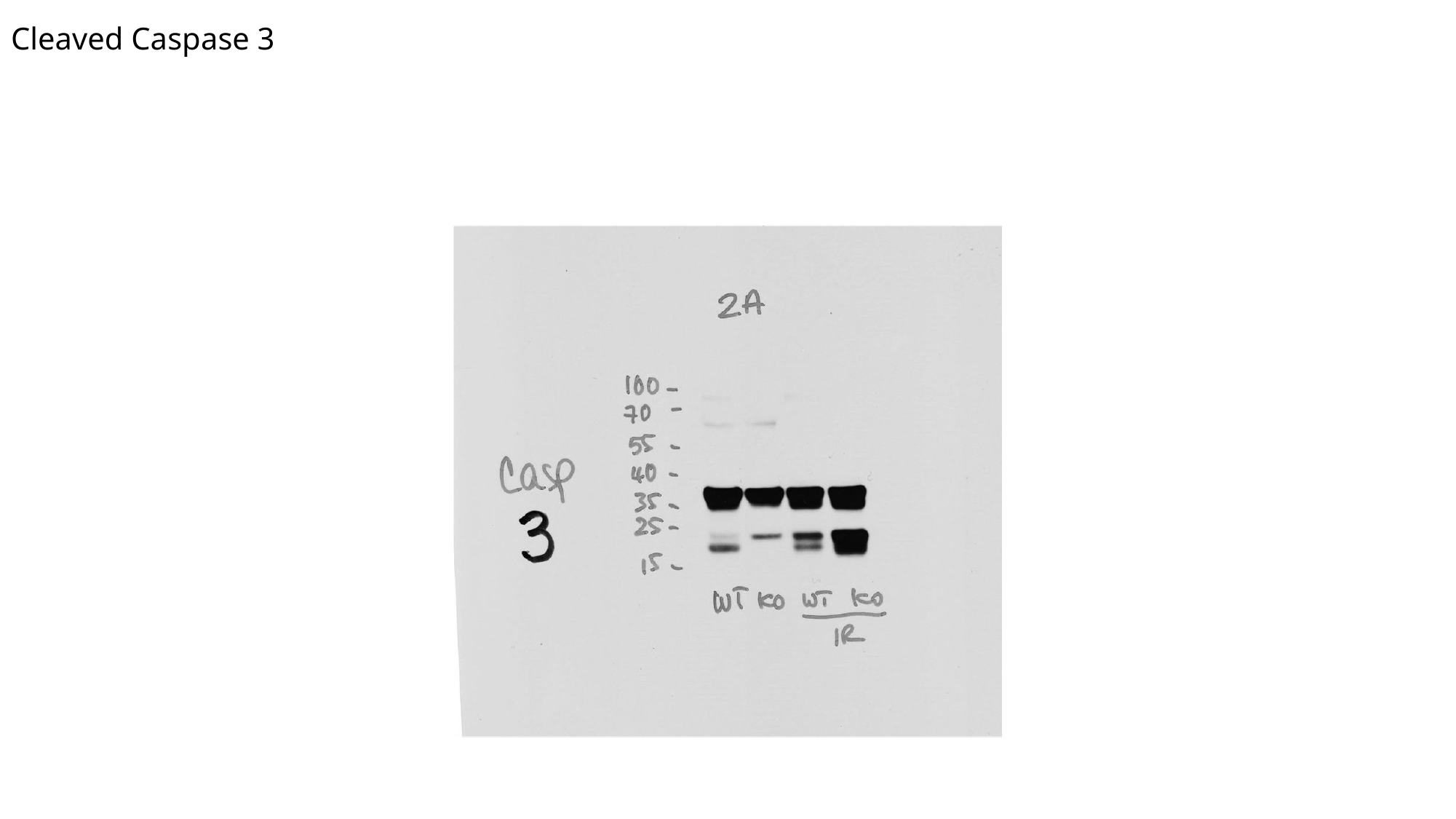

# Cleaved Caspase 3

## Slide 4
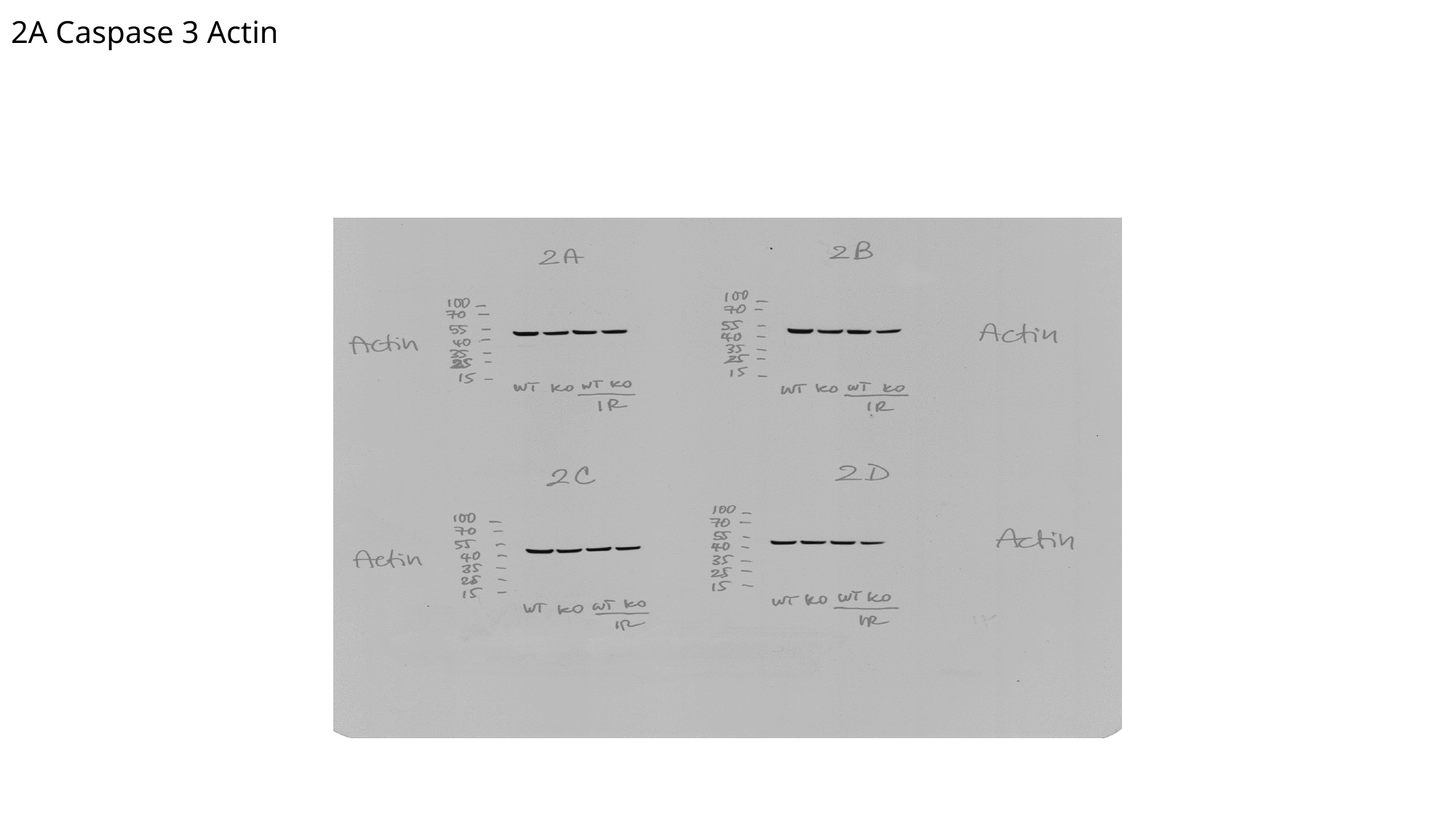

# 2A Caspase 3 Actin

## Slide 5
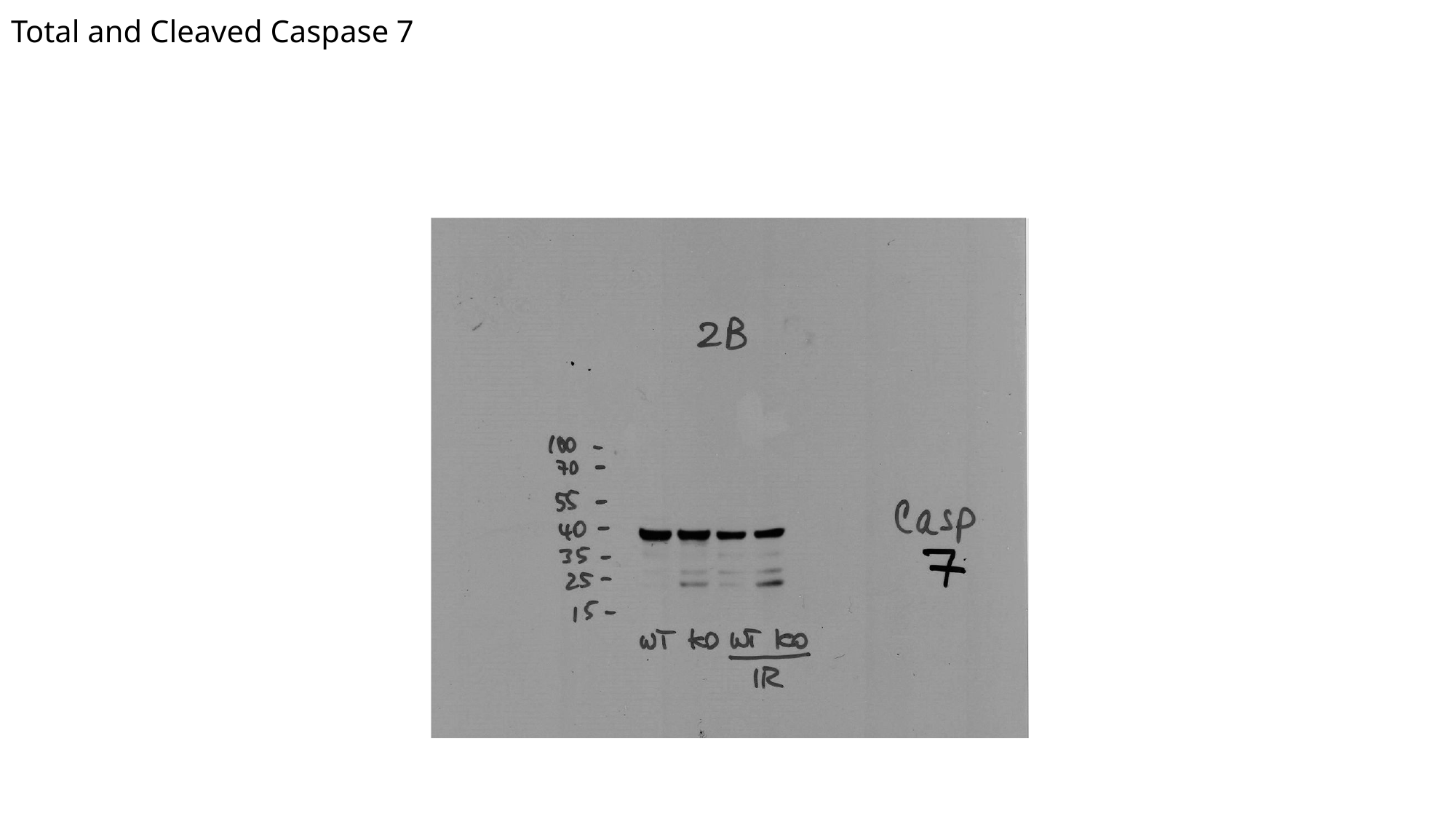

# Total and Cleaved Caspase 7

## Slide 6
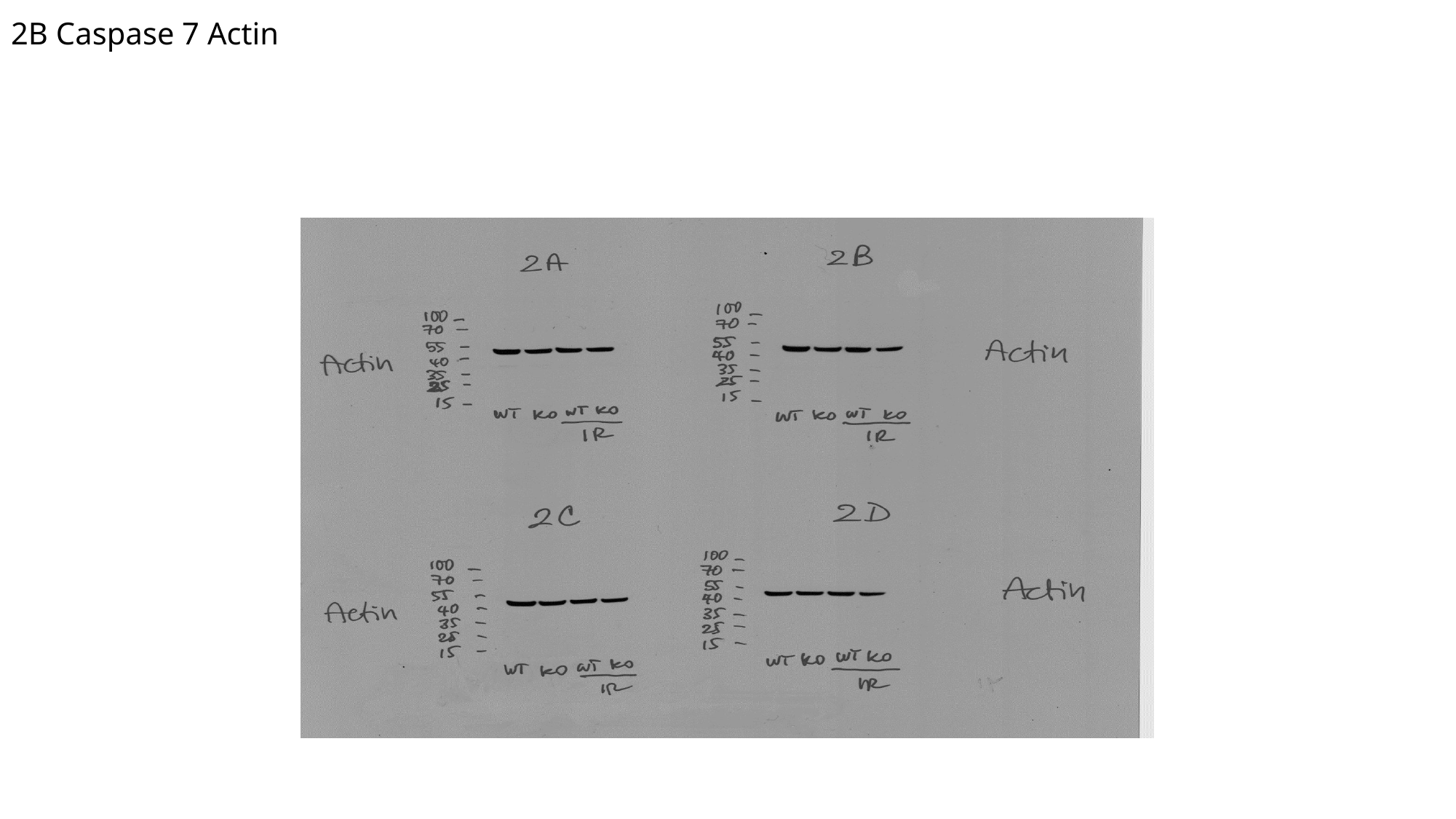

# 2B Caspase 7 Actin

## Slide 7
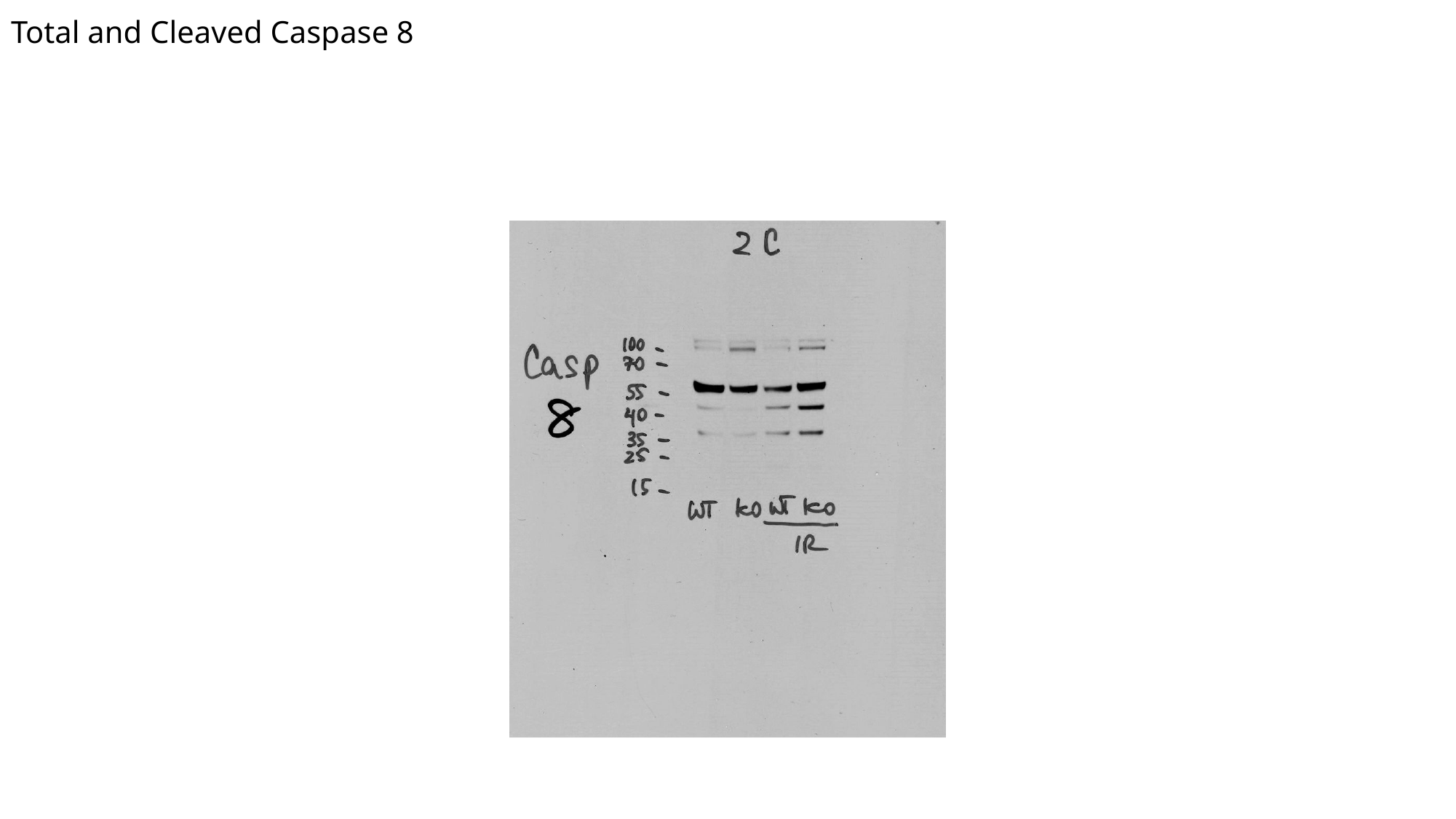

# Total and Cleaved Caspase 8

## Slide 8
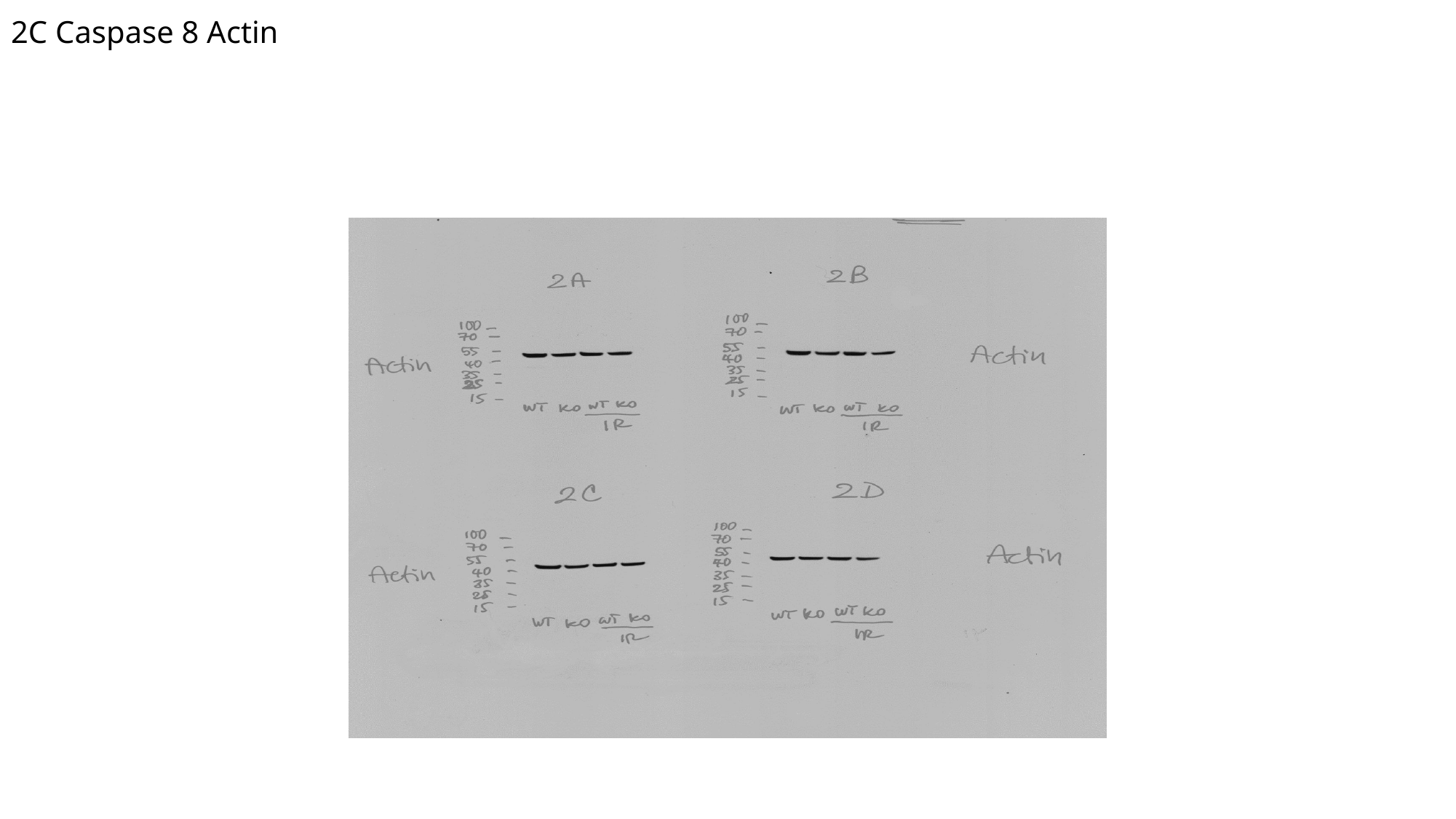

# 2C Caspase 8 Actin

## Slide 9
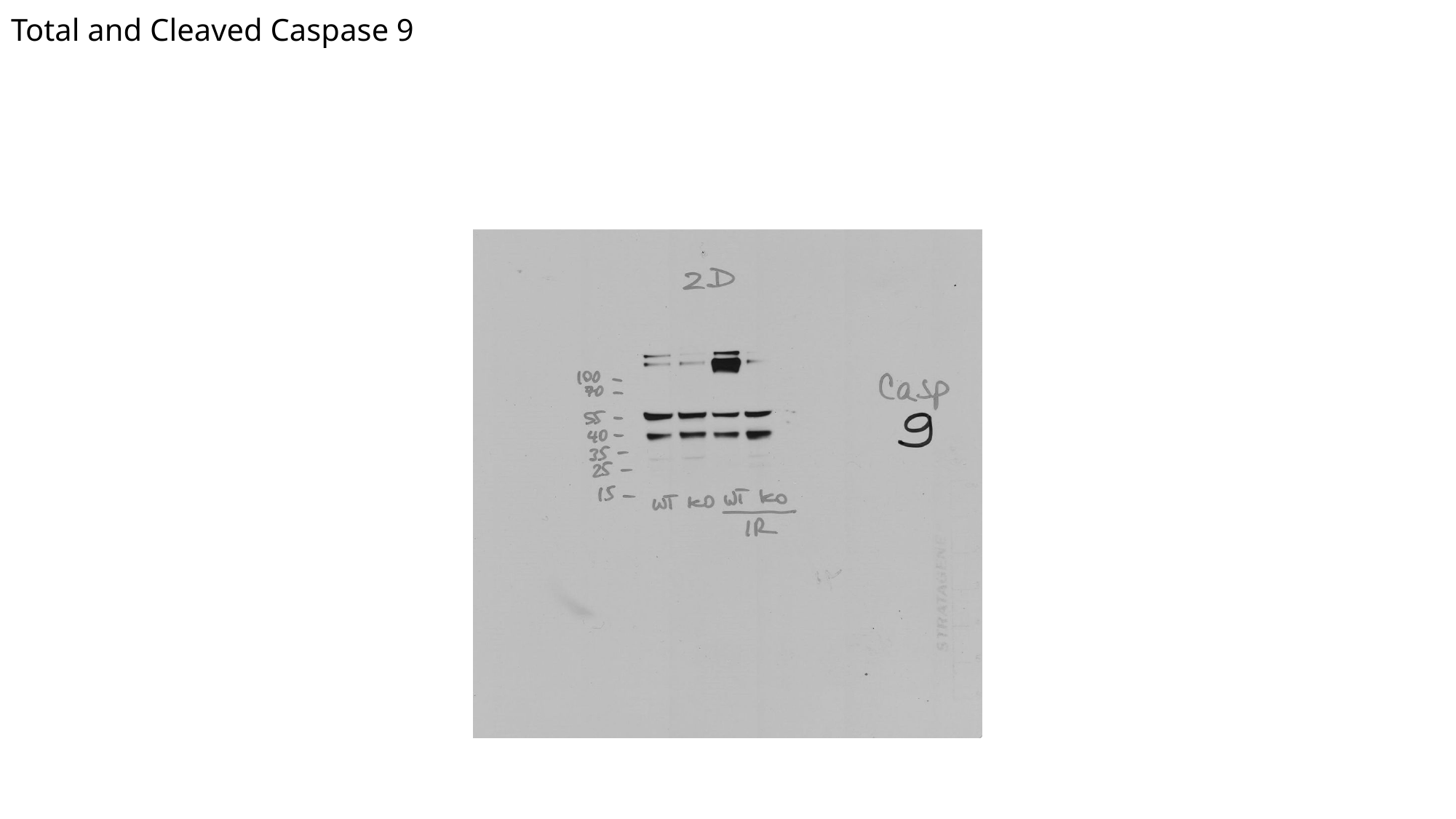

# Total and Cleaved Caspase 9

## Slide 10
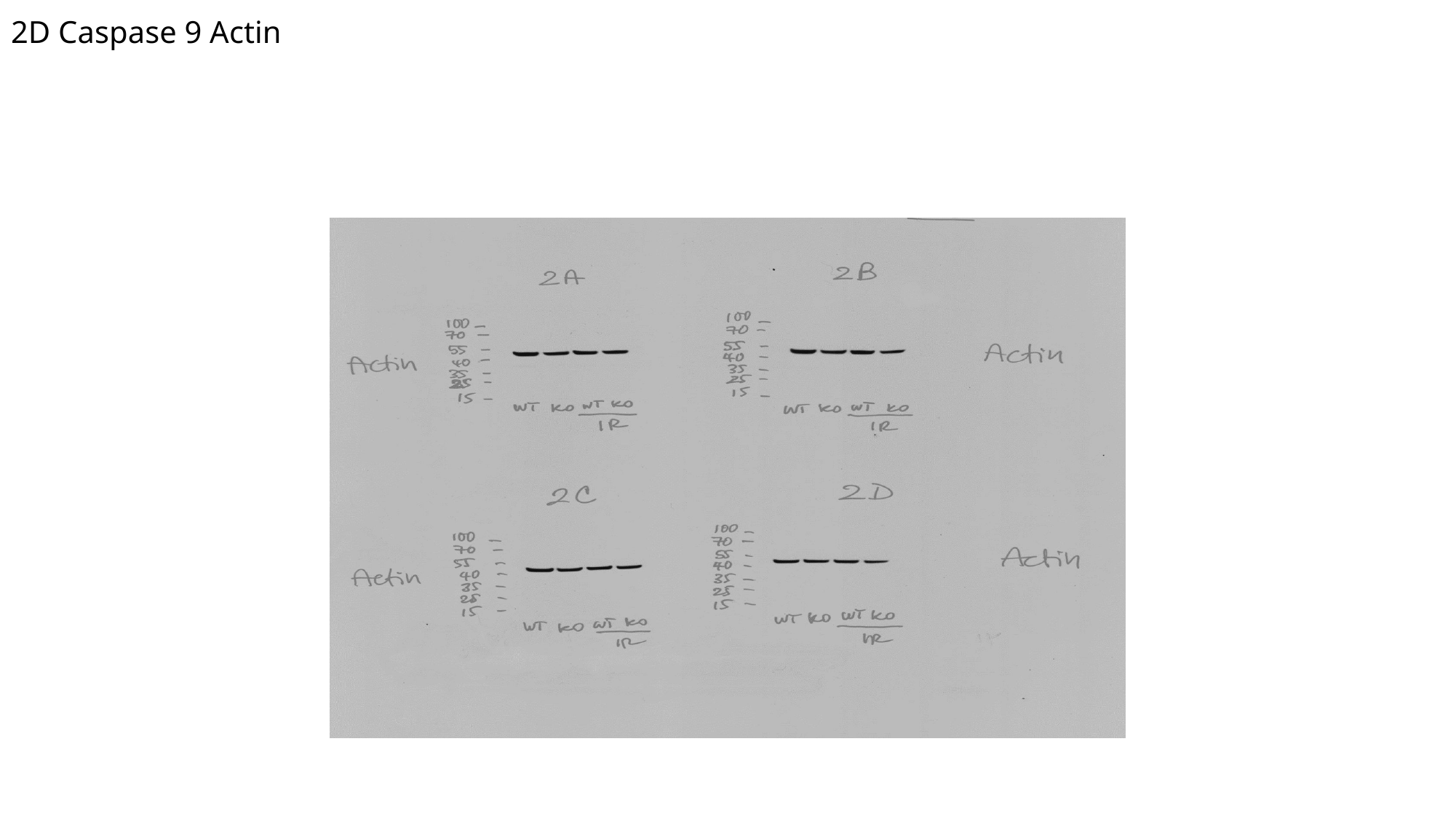

# 2D Caspase 9 Actin

## Slide 11
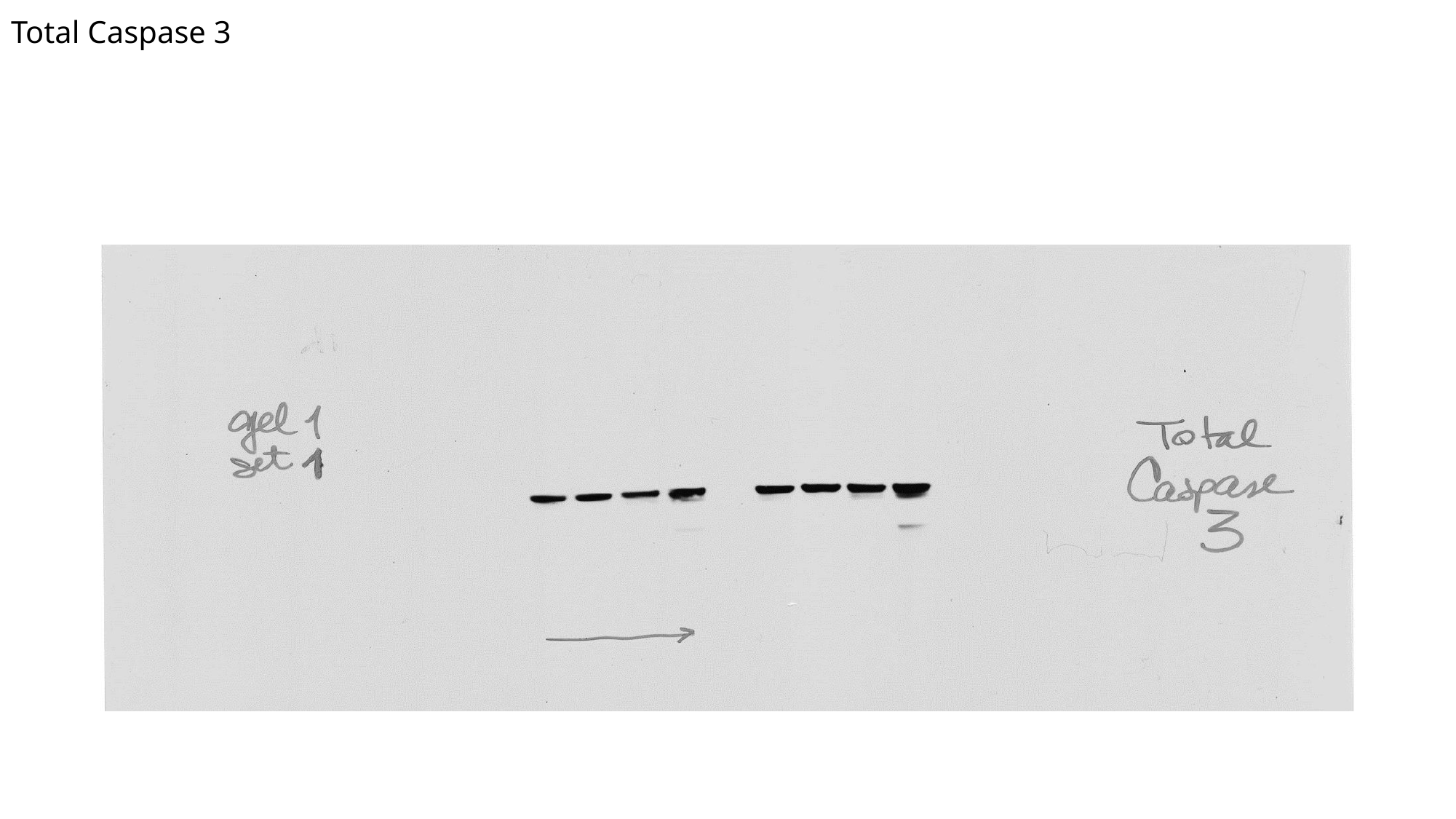

# Total Caspase 3

## Slide 12
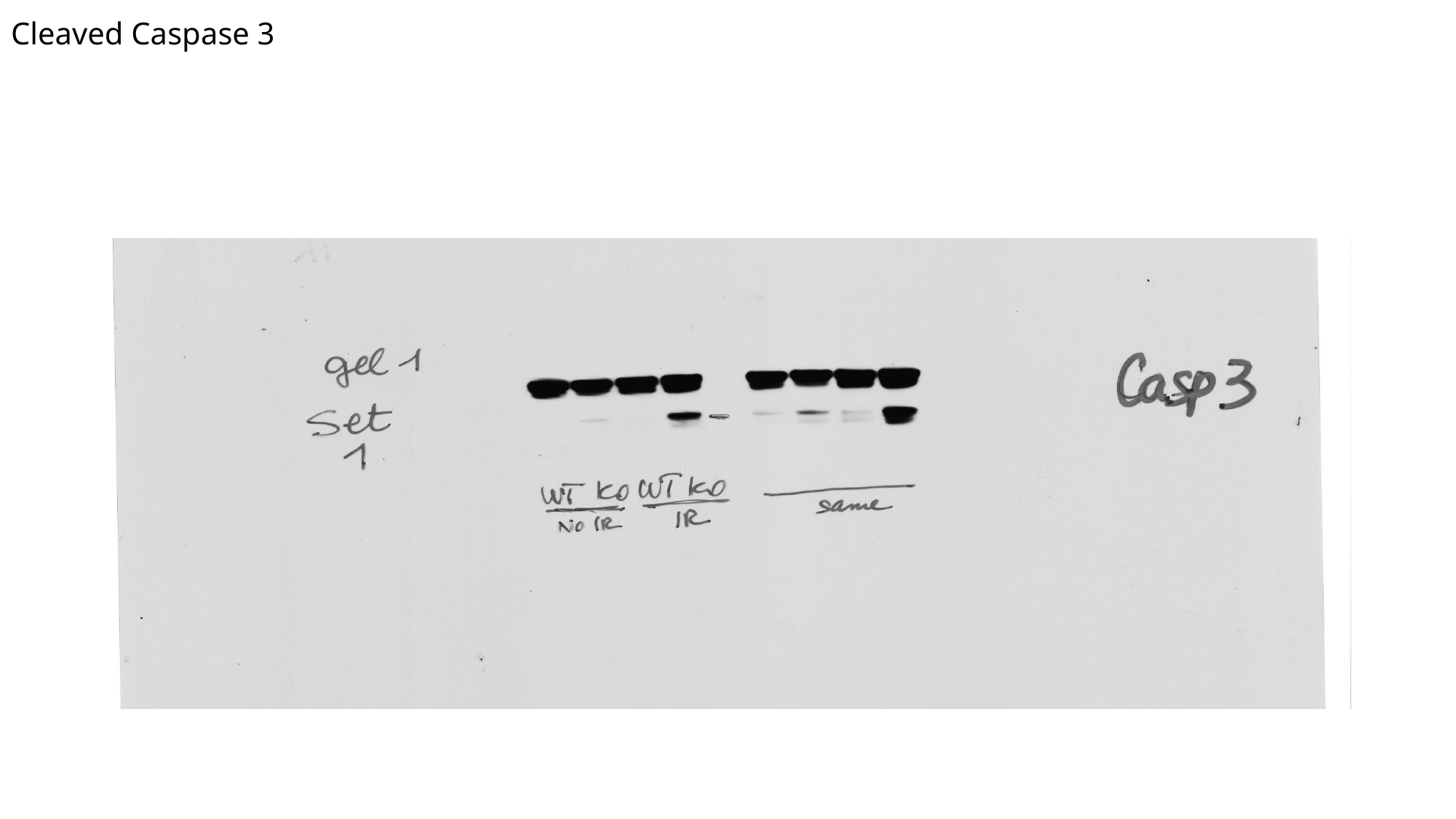

# Cleaved Caspase 3

## Slide 13
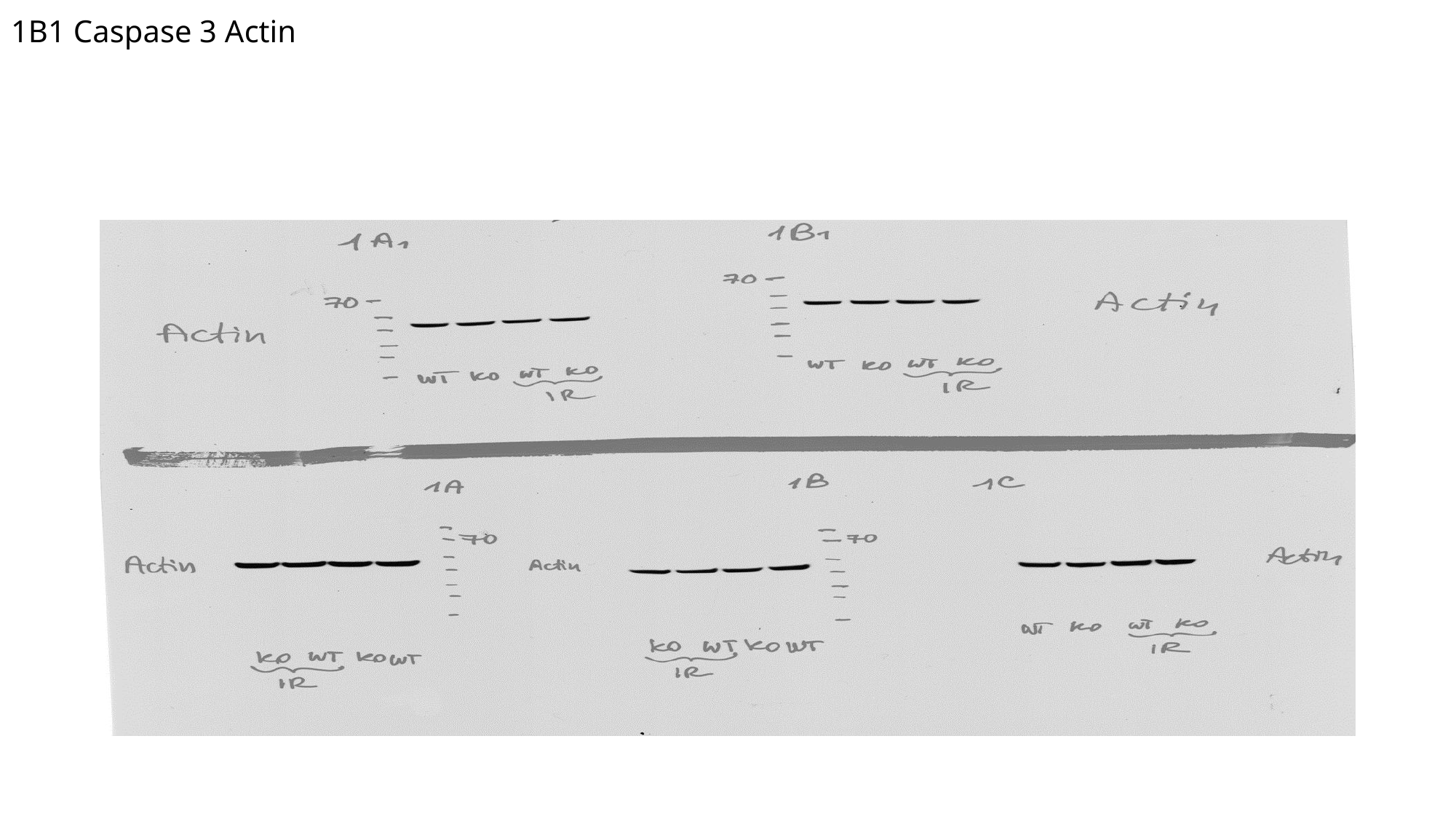

# 1B1 Caspase 3 Actin

## Slide 14
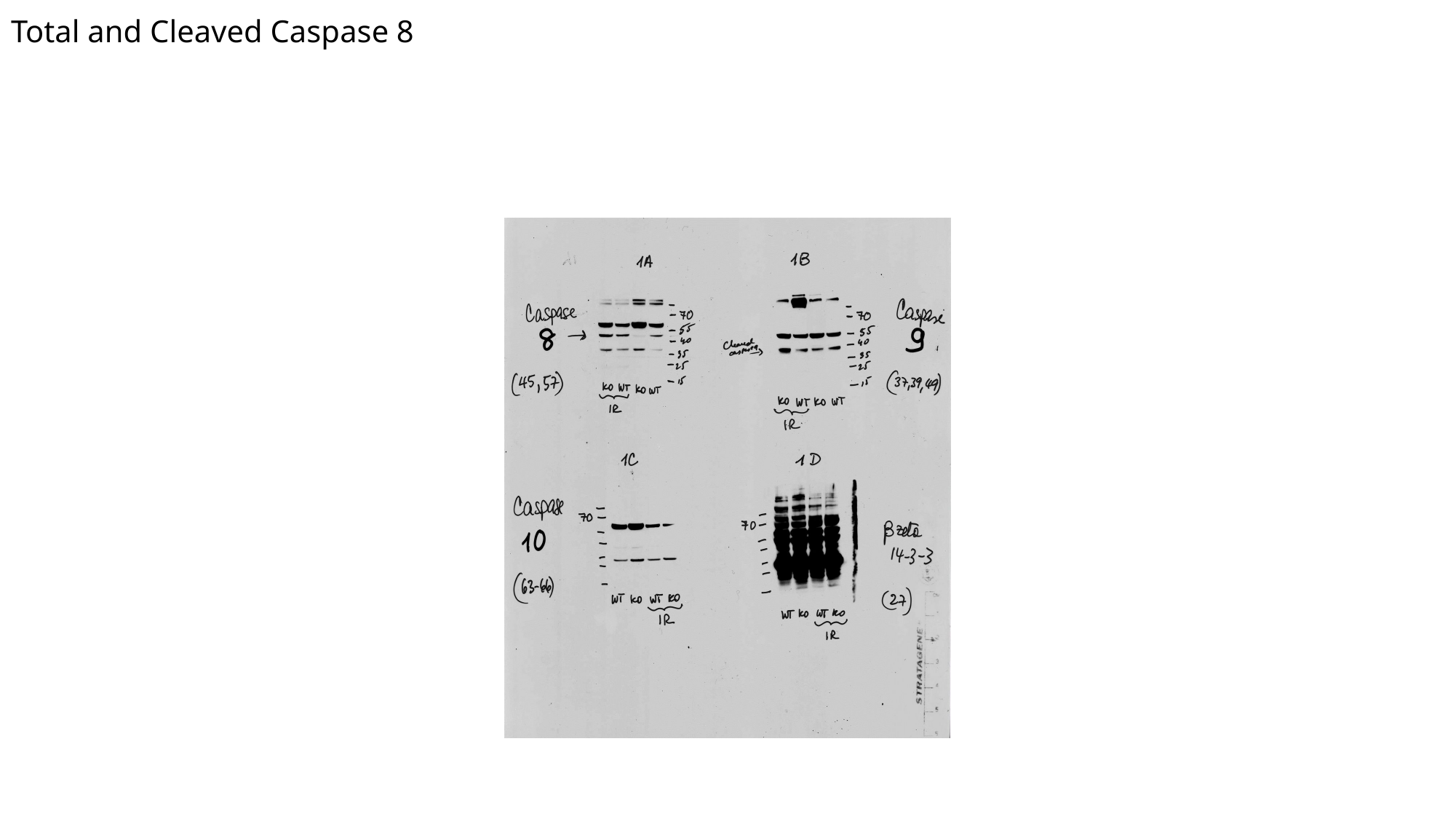

# Total and Cleaved Caspase 8

## Slide 15
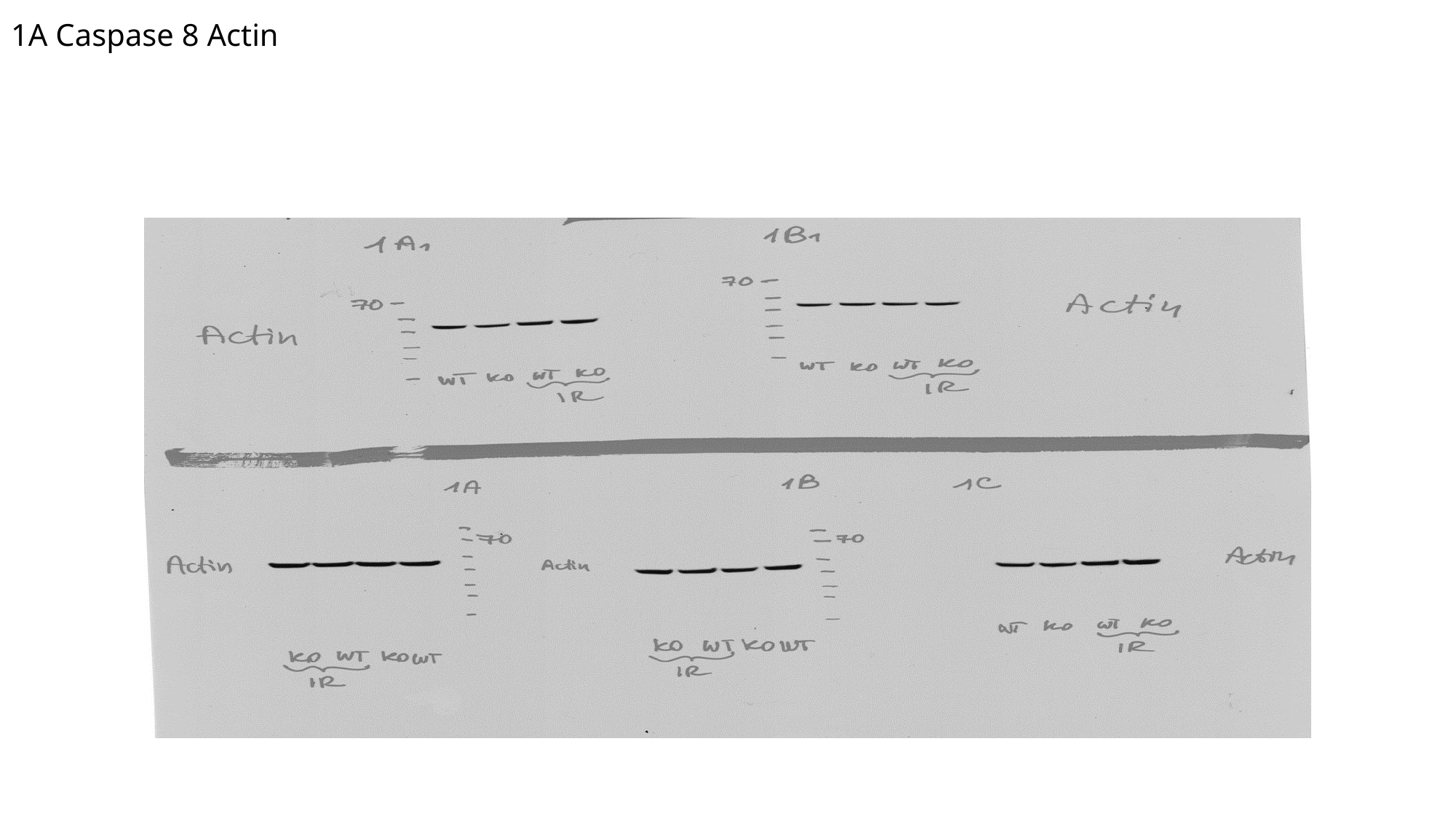

# 1A Caspase 8 Actin

## Slide 16
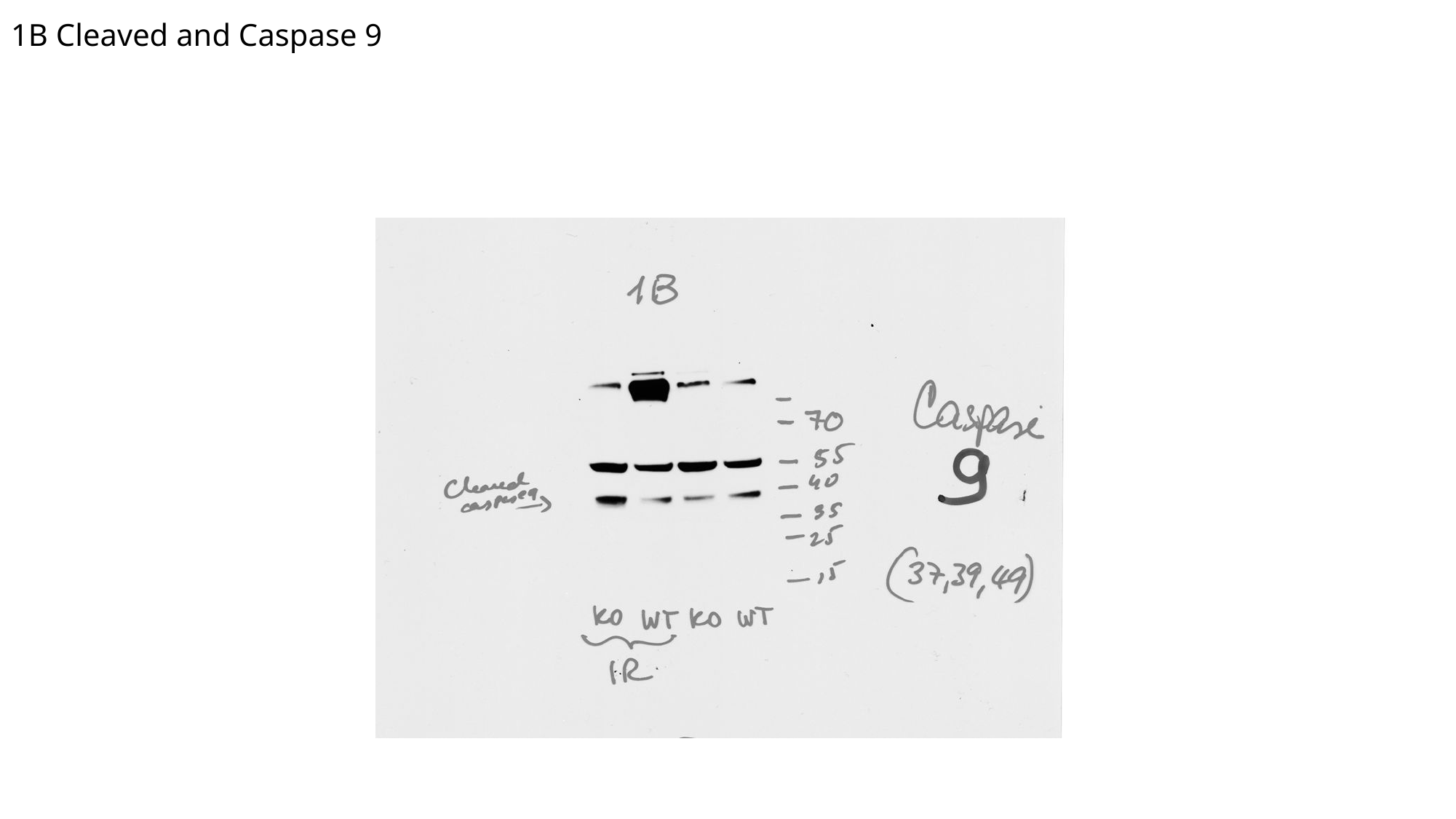

# 1B Cleaved and Caspase 9

## Slide 17
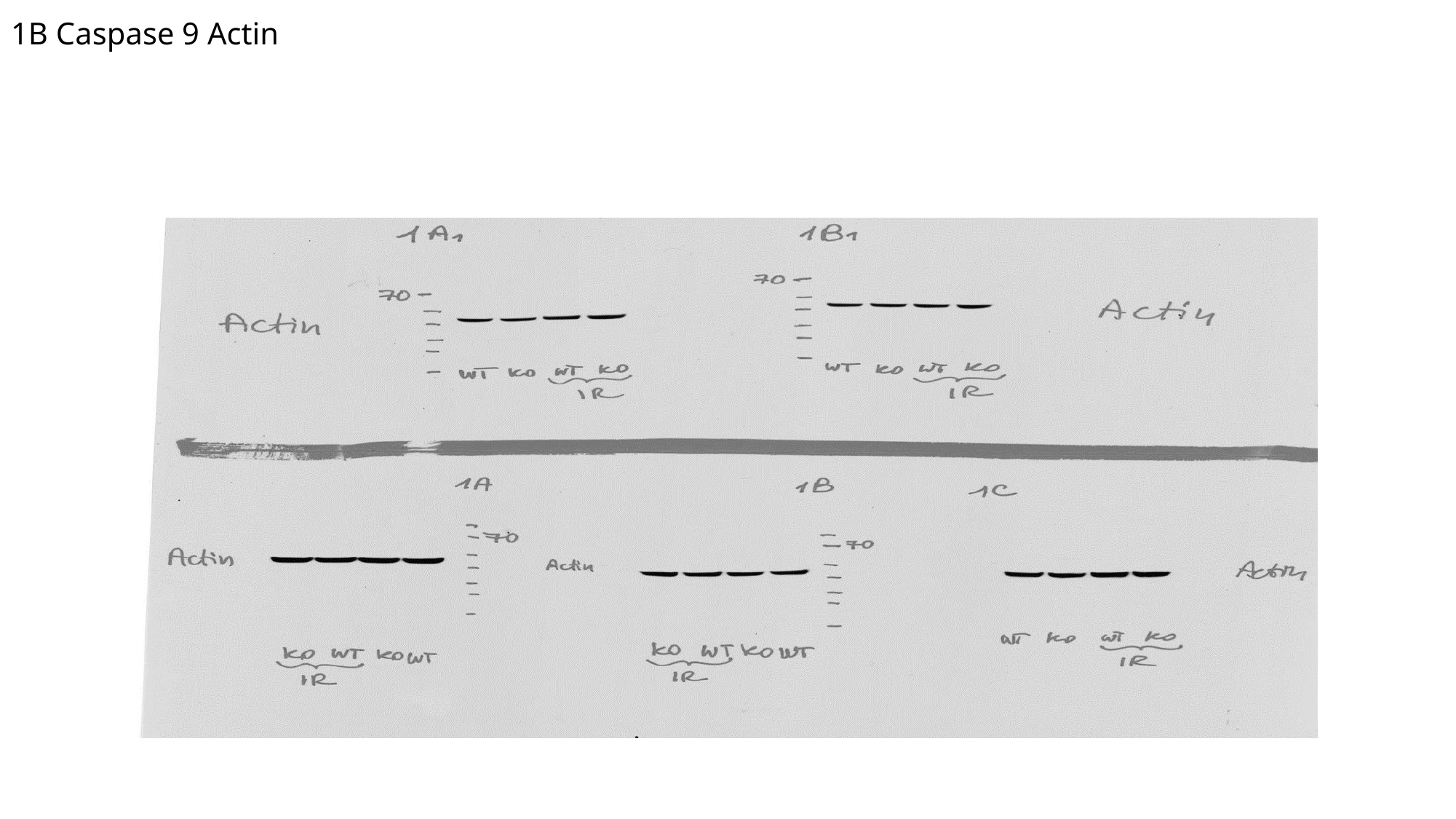

# 1B Caspase 9 Actin

## Slide 18
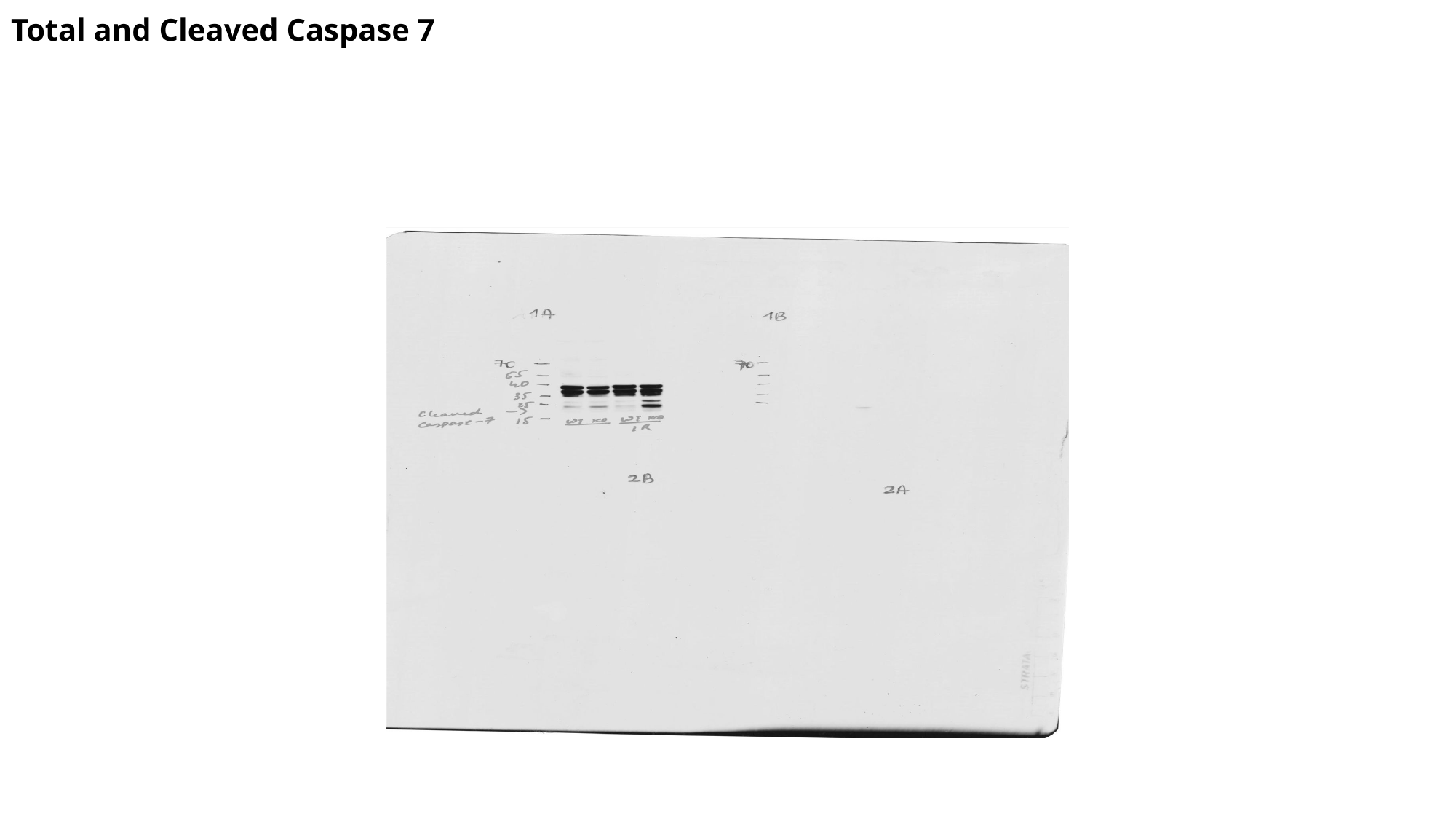

# Total and Cleaved Caspase 7

## Slide 19
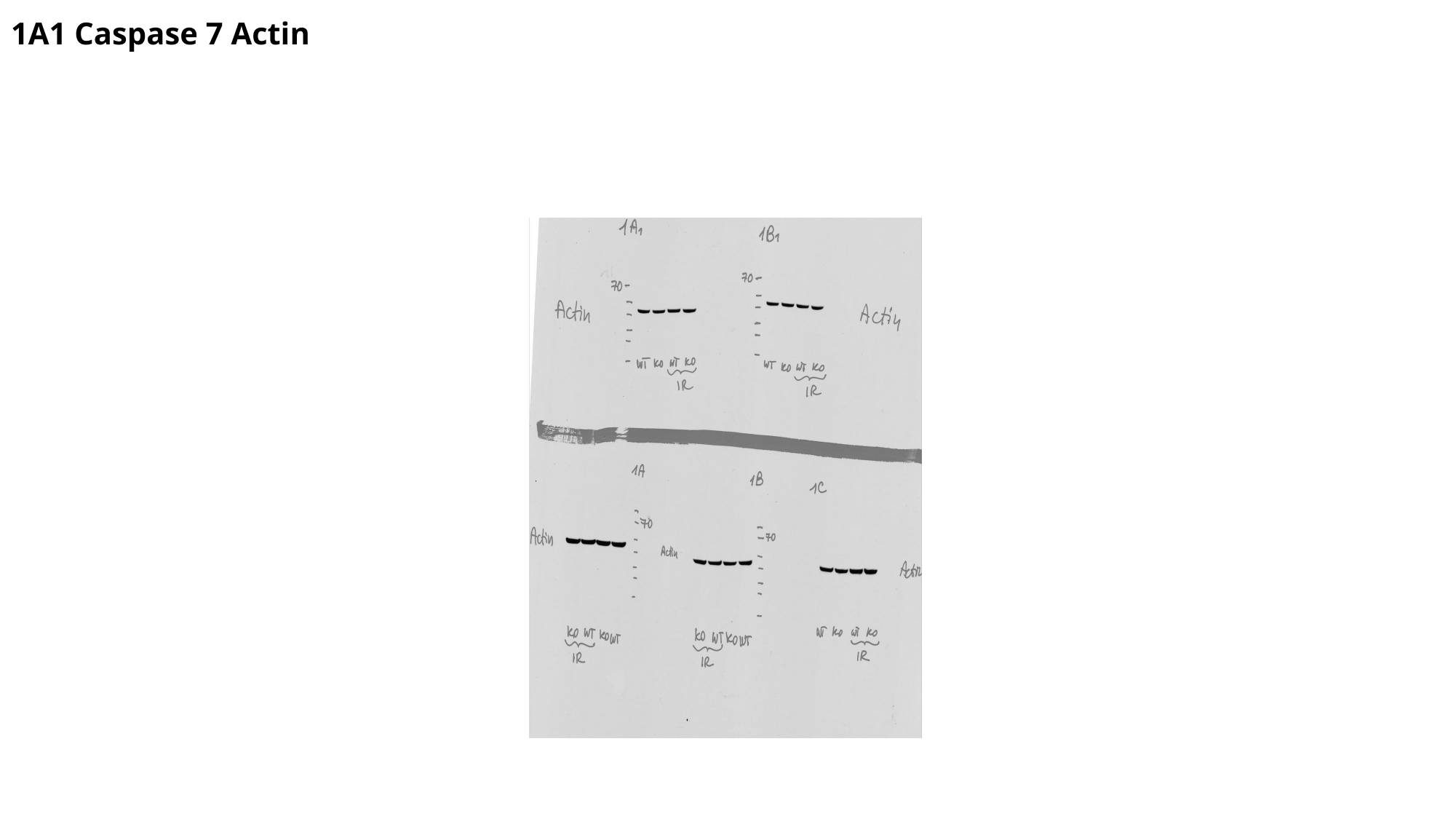

# 1A1 Caspase 7 Actin

## Slide 20
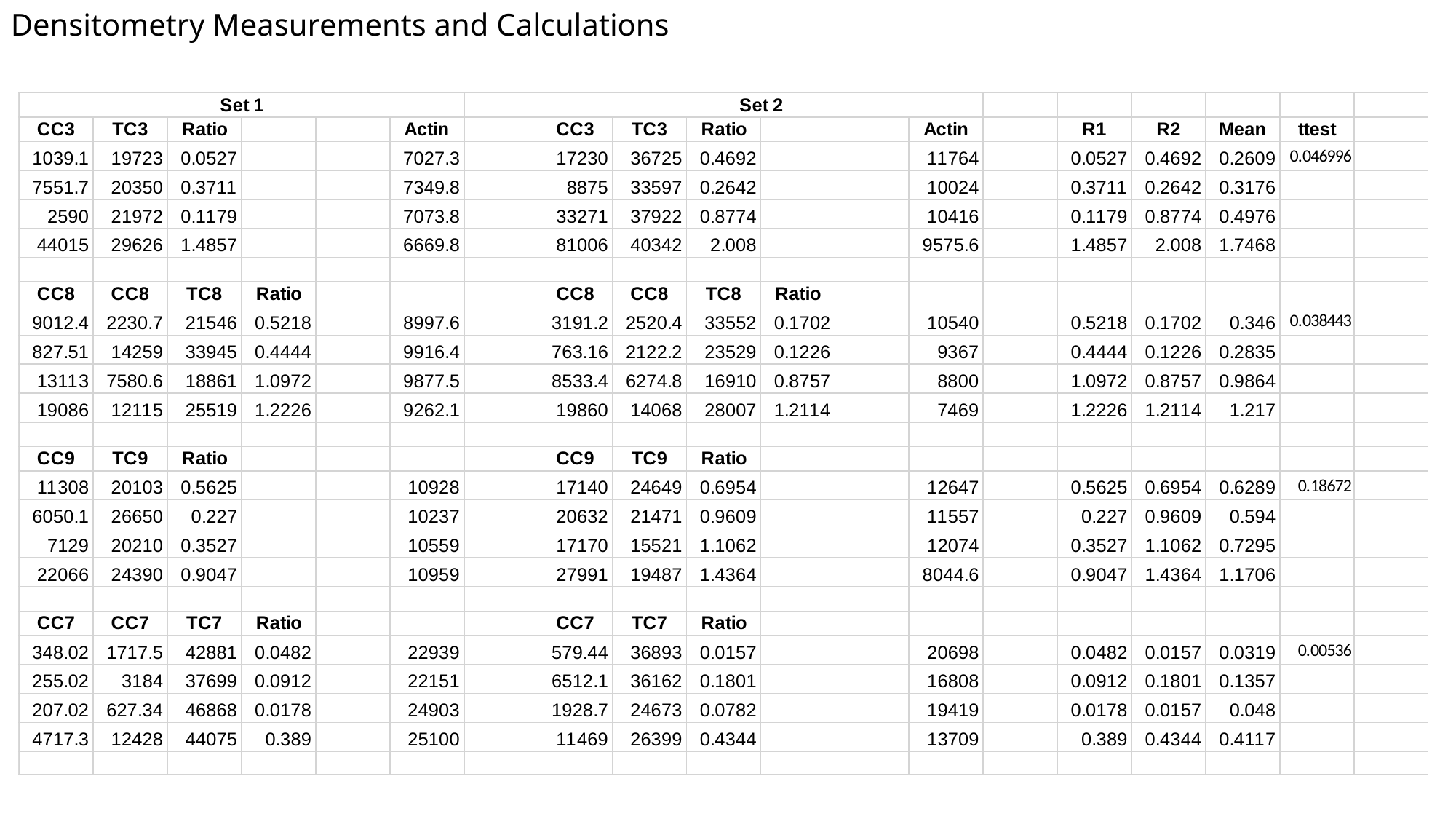

Densitometry Measurements and Calculations
